# Hyperactive STAT5 hijacks T cell receptor signaling and drives immature T cell acute lymphoblastic leukemia

**DOI:** 10.1172/JCI168536

**Published:** 2024-04-15

**Authors:** Tobias Suske, Helena Sorger, Gabriele Manhart, Frank Ruge, Nicole Prutsch, Mark W. Zimmerman, Thomas Eder, Diaaeldin I. Abdallah, Barbara Maurer, Christina Wagner, Susann Schönefeldt, Katrin Spirk, Alexander Pichler, Tea Pemovska, Carmen Schweicker, Daniel Pölöske, Emina Hubanic, Dennis Jungherz, Tony Andreas Müller, Myint Myat Khine Aung, Anna Orlova, Ha Thi Thanh Pham, Kerstin Zimmel, Thomas Krausgruber, Christoph Bock, Mathias Müller, Maik Dahlhoff, Auke Boersma, Thomas Rülicke, Roman Fleck, Elvin Dominic de Araujo, Patrick Thomas Gunning, Tero Aittokallio, Satu Mustjoki, Takaomi Sanda, Sylvia Hartmann, Florian Grebien, Gregor Hoermann, Torsten Haferlach, Philipp Bernhard Staber, Heidi Anne Neubauer, Alfred Thomas Look, Marco Herling, Richard Moriggl

**Affiliations:** 1 Institute of Animal Breeding and Genetics and; 2 Institute for Medical Biochemistry, University of Veterinary Medicine Vienna, Vienna, Austria.; 3 Department of Pediatric Oncology, Dana-Farber Cancer Institute, Harvard Medical School, Boston, Massachusetts, USA.; 4 Department of Chemical and Physical Sciences, University of Toronto Mississauga, Mississauga, Ontario, Canada.; 5 Department of Chemistry, University of Toronto, Toronto, Ontario, Canada.; 6 Department of Medicine I, Clinical Division of Hematology, Medical University of Vienna, Vienna, Austria.; 7 Department I of Internal Medicine, Center for Integrated Oncology, Aachen-Bonn-Cologne-Duesseldorf, University of Cologne, Cologne, Germany.; 8 CeMM Research Center for Molecular Medicine of the Austrian Academy of Sciences, Vienna, Austria.; 9 Institute of Artificial Intelligence, Center for Medical Data Science, Medical University of Vienna, Vienna, Austria.; 10 Institute of in vivo and in vitro Models, University of Veterinary Medicine Vienna, Vienna, Austria.; 11 Ludwig Boltzmann Institute for Hematology and Oncology, Medical University of Vienna, Vienna, Austria.; 12 Janpix, London, United Kingdom.; 13 Institute for Molecular Medicine Finland, Helsinki Institute of Life Science, University of Helsinki, Helsinki, Finland.; 14 iCAN Digital Precision Cancer Medicine Flagship, Helsinki, Finland.; 15 Department of Cancer Genetics, Institute for Cancer Research, Oslo University Hospital, Oslo, Norway.; 16 Oslo Centre for Biostatistics and Epidemiology, Faculty of Medicine, University of Oslo, Oslo, Norway.; 17 Hematology Research Unit Helsinki, University of Helsinki and Helsinki University Hospital Comprehensive Cancer Center, Helsinki, Finland.; 18 Translational Immunology Research Program and Department of Clinical Chemistry and Hematology, University of Helsinki, Helsinki, Finland.; 19 Cancer Science Institute of Singapore and Department of Medicine, Yong Loo Lin School of Medicine, National University of Singapore, Singapore.; 20 Dr. Senckenberg Institute of Pathology, Goethe University, Frankfurt am Main, Germany.; 21 St. Anna Children’s Cancer Research Institute, Vienna, Austria.; 22 MLL Munich Leukemia Laboratory, Munich, Germany.; 23 Department of Hematology, Cellular Therapy and Hemostaseology, University of Leipzig, Leipzig, Germany.; 24 Department of Biosciences and Medical Biology, Paris Lodron University of Salzburg, Salzburg, Austria.

**Keywords:** Genetics, Hematology, Leukemias, T cell development, T cell receptor

## Abstract

T cell acute lymphoblastic leukemia (T-ALL) is an aggressive immature T cell cancer. Mutations in *IL7R* have been analyzed genetically, but downstream effector functions such as STAT5A and STAT5B hyperactivation are poorly understood. Here, we studied the most frequent and clinically challenging STAT5B^N642H^ driver in T cell development and immature T cell cancer onset and compared it with STAT5A hyperactive variants in transgenic mice. Enhanced STAT5 activity caused disrupted T cell development and promoted an early T cell progenitor–ALL phenotype, with upregulation of genes involved in T cell receptor (TCR) signaling, even in absence of surface TCR. Importantly, TCR pathway genes were overexpressed in human T-ALL and mature T cell cancers and activation of TCR pathway kinases was STAT5 dependent. We confirmed STAT5 binding to these genes using ChIP-Seq analysis in human T-ALL cells, which were sensitive to pharmacologic inhibition by dual STAT3/5 degraders or ZAP70 tyrosine kinase blockers in vitro and in vivo. We provide genetic and biochemical proof that STAT5A and STAT5B hyperactivation can initiate T-ALL through TCR pathway hijacking and suggest similar mechanisms for other T cell cancers. Thus, STAT5 or TCR component blockade are targeted therapy options, particularly in patients with chemoresistant clones carrying STAT5B^N642H^.

## Abstract 2

Hyperactive STAT5A or STAT5B initiates thymic T-cell development and transformation to T-cell acute lymphoblastic leukemia.

## Introduction

Acute lymphoblastic leukemia (ALL) is subdivided into B and T cell acute lymphoblastic leukemia (T-ALL), of which T-ALL represents up to 15% of pediatric and 25% of adult ALL cases (1, 2). The disease is characterized by the malignant expansion of immature T cells harboring chromosomal rearrangements that lead to activation of oncogenic transcription factors, e.g., TAL1/2, TLX1/3, LMO1/2, or HOXA (3, 4). These rearrangements often involve translocations to T cell receptor (TCR) gene enhancers, promoting their aberrant expression (3). This induces self-renewal, T cell differentiation arrest and transformation through accumulation of further mutations (5, 6), like gain-of-function (GOF) *NOTCH1* mutations, losses of tumor suppressor gene functions (e.g., *PTEN*, *CDKN2A*, *TP53*), or inactivation of genes encoding epigenetic regulators (e.g., *EZH2*, *SUZ12*, *EED*) (3, 4, 7). Furthermore, GOF mutations in *IL7R*, *JAK1*, *JAK3*, or *STAT5B* are found in more than 30% of pediatric/adult T-ALL cases (4, 8–11). Particularly, serine-to-cysteine substitutions or mutations in the transmembrane domain of the IL-7 receptor (IL-7R) α chain are well characterized and known to induce constitutive JAK1-STAT5 activation (12–14). Moreover, overexpression of IL-7R or inactivation of phosphatases such as CD45 can lead to activation of the IL-7R/JAK1/JAK3/STAT5B pathway in T-ALL (8, 12). STAT5A and STAT5B are strongly related, showing approximately 92% amino acid identity and approximately 94% similarity (15). STAT5B is described as a stronger oncoprotein compared with STAT5A, but both display redundant transcriptional activity through heterodimerization (16–19). Importantly, *STAT5B^N642H^* is a recurrent, activating hotspot mutation found in T cell cancers, including several mature leukemias and lymphomas, as well as NK cell and myeloid neoplasms (19–23). The aggressive nature of aberrant STAT5 activity is illustrated by transgenic mice expressing STAT5B^N642H^ or STAT5A^S710F^, which develop peripheral T cell lymphoma/leukemia (PTCL), with dominant differentiation toward CD8^+^ effector memory T cells (16–18). *STAT5B^N642H^* is the most frequent *STAT5B* mutation in T-ALL or in phenotypically similar T cell lymphoblastic lymphoma (T-LBL), both diseases primarily affecting children (4, 18, 19, 24, 25). Here, we use T-ALL to refer to cases of T-ALL and T-LBL; this is supported by the fact that no clinical distinction between T-ALL and T-LBL exists for murine tumors. Despite advances in chemotherapy treatment, therapy resistance remains the major challenge in combating T-ALL (26). The presence of *STAT5B^N642H^* detected by sequencing efforts was found to correlate with worse prognosis and a higher incidence of relapse upon chemotherapy in a cohort of 301 patients with T-ALL (24). Moreover, *JAK1*/*JAK3*/*STAT5B* GOF mutations accumulated in expanded clones of T-ALL, defining them as potential oncogenes (27). However, these mutations are genetically still poorly evaluated.

Complete loss of STAT5 is embryonically lethal because of defective erythropoiesis in mice. The few (~2%) homozygous STAT5-null survivor mice displayed approximately 98% reduction in thymocyte numbers (28). This emphasizes the fact that both STAT5A and STAT5B are essential for T cell expansion and survival during mammalian development, in particular for T cell differentiation (28). Studies have revealed that the IL-7R/JAK1/3/STAT5 axis is essential for development of different thymic T cell subsets, inducing transcription of cell cycle progression genes, BCL-2 family members, or transcription factors such as RUNX3 or FOXP3 (29, 30). Particularly, common γ chain (γ_c_) signaling governs CD8^+^ lineage commitment via STAT5, and its conditional deletion in DP thymocytes reduced the frequency of SP8 cells (31). However, it is currently unknown, how *STAT5A* or *STAT5B* GOF mutations affect thymic development and, ultimately, induce transformation of immature T cells to T-ALL in presence or absence of TCR surface chain expression.

Here, we show that mutation-driven hyperactivation of STAT5A or STAT5B promotes T-ALL without the requirement of upstream GOF mutations. STAT5B^N642H^ especially caused drastic changes in thymic maturation, architecture, and global gene transcription. Mechanistically, we found that hyperactive STAT5 promotes an activated T cell phenotype and drives expression of kinases that are normally involved in TCR signaling. STAT5B^N642H^ or STAT5A^S710F^ both acted as oncoproteins in a redundant fashion to induce T-ALL, but the clinically relevant STAT5B^N642H^ caused more aggressive phenotypes. We suggest that these findings are also highly relevant for other T cell cancers. Blocking STAT3/5 and ZAP70 individually or in combination impaired T-ALL growth/survival. We conclude that STAT5 and TCR-ligated tyrosine kinases represent valuable pharmacologic targets in T-ALL with high IL-7R/JAK1/3/STAT5 activation.

## Results

### STAT5B^N642H^ is recurrently mutated in patients with T-ALL and alters T cell development.

**Figure 1:**
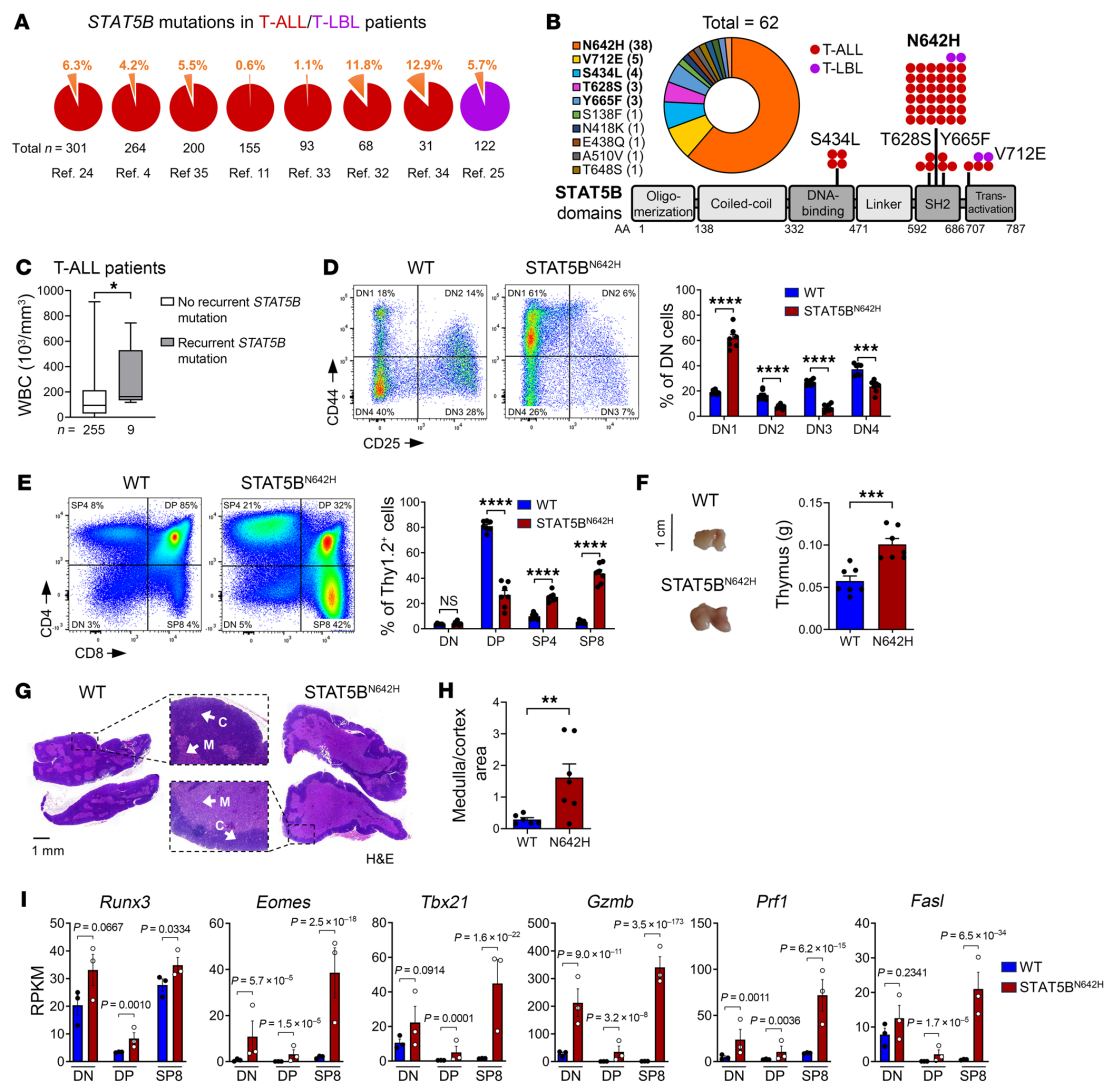
STAT5B^N642H^ occurs in patients with T-ALL and impacts thymic development and architecture. (**A**) Percentages of patients with somatic *STAT5B* mutations found in 8 studies (24, 4, 35, 11, 33, 32, 34, 25) in T-ALL and T-LBL. (**B**) Incidence and localization in the STAT5 protein with amino acid (AA) position of respective somatic mutations in *STAT5B* found in 1,234 patients with T-ALL or T-LBL from the studies indicated in **A**. (**C**) White blood cell (WBC) counts of patients with or without recurrent *STAT5B* mutations. Data were extracted from ref. 4. (**D** and **E**) Flow cytometry analysis and percentages of DN1, DN2, DN3, and DN4 cells as well as DN, DP, SP4, and SP8 cells from STAT5B^N642H^ mice (*n* = 7) and WT littermates (*n* = 7) gated on Thy1.2^+^ thymocytes. Two independent experiments were performed. (**F**) Representative images and thymus weights in g of 8-week-old WT (*n* = 7) or STAT5B^N642H^ (*n* = 7) mice. (**G**) Representative H&E-stained whole thymi from WT or STAT5B^N642H^ mice, indicating cortical (C) and medullary (M) regions. Original magnification: ×10 (boxed by dashed lines); scale bar: 1 mm. (**H**) Quantification of medullary-cortical area ratios with *n* = 6 WT and *n* = 7 STAT5B^N642H^ using the ImageJ (NIH) software. (**I**) RPKM values of *Runx3*, *Eomes*, *Tbx21*, *Gzmb*, *Prf1*, and *Fasl* in DN, DP, and SP8 stages of WT or STAT5B^N642H^ thymocytes. In **C**–**F** and **H**, significant differences are indicated as *P < 0.05, **P < 0.01, ***P < 0.001, ****P < 0.0001 by unpaired 2-tailed Student’s *t* test. Data are shown as the mean ± SEM. In **I**, adjusted *P* values (*P*_adj_) of respective comparisons were taken from DESeq2 analysis. Data are shown as the mean ± SEM.

GOF mutations in *IL-7R*, *JAK1*, *JAK3*, or *STAT5B* are found in up to 30% of patients with T-ALL (3). We data-mined 8 independent studies with sequencing data from 1,234 patients with T-ALL (4, 11, 24, 25, 32–35) and found that a mean of 3.8% (0.6%–12.9%) of patients harbored *STAT5B* mutations (Figure 1A). *STAT5B^N642H^* was the most frequent recurrent mutation, accounting for 38 of the 62 (61.3%) cases with *STAT5B* mutations (Figure 1B). Five of 14 different *STAT5B* mutations have been detected in ≥3 patients with T-ALL (Figure 1B), and these patients had almost twice as high white blood cell (WBC) counts as patients with unmutated *STAT5B*, suggesting that STAT5B hyperactivation correlates with higher leukemic burden and extramedullary cancer cell survival (Figure 1C).

To investigate mechanistic consequences of STAT5B^N642H^ expression on T cell transformation, we analyzed transgenic mice expressing human STAT5B^N642H^ under the control of the *Vav1* promoter, driving transgene expression from the hematopoietic stem cell level onward, including lymphoid progenitors (36). STAT5B^N642H^ mice succumb to disease at approximately 10 weeks of age due to mature, postthymic CD8^+^ T cell neoplasia (16).

Thymic T cell development undergoes CD4/CD8 double-negative (DN) to double-positive (DP) transition prior to CD4 or CD8 single-positive (SP4/SP8) lineage commitment (37). STAT5B^N642H^ mice exhibited progressing disease associated with increased spleen weight (Supplemental Figure 1A; supplemental material available online with this article; https://doi.org/10.1172/JCI168536DS1). Increased STAT5 phosphorylation (pY-STAT5) in thymi of diseased STAT5B^N642H^ mice was confirmed by Western blotting (Supplemental Figure 1B). Next, we determined DN1 (CD25^–^CD44^+^), DN2 (CD25^+^CD44^+^), DN3 (CD25^+^CD44^–^), DN4 (CD25^–^CD44^–^), DP, SP4, and SP8 populations by flow cytometry. We found that STAT5B^N642H^ promoted approximately 3-fold expansion of the most immature DN1 cells, at the expense of DN2, 3, and 4 populations, compared with WT littermates at 8 weeks of age (Figure 1D). DP cells were 3-fold reduced, while SP4 and SP8 cells were approximately 2.5- and approximately 8-fold expanded in STAT5B^N642H^ mice (Figure 1E). We found elevated levels of CD69 in DP thymocytes of STAT5B^N642H^ mice, suggesting an advantage in positive selection. Conversely, CD69 levels were reduced in SP4 and SP8 cells from STAT5B^N642H^ mice, implying facilitated commitment to SP4 and SP8 lineages and a mature SP stage to facilitate thymic egress (38, 39) (Supplemental Figure 1C). Surface CD5 was reduced in STAT5B^N642H^ SP cells compared with that in WT controls (Supplemental Figure 1D), which suggests a lower inhibitory effect on TCR signaling due to reduced binding to CD3ζ-ZAP70 components of the TCR/CD3 complex (40–42). These effects on thymic development in the STAT5B^N642H^ mice were already apparent at 4 and 6 weeks of age to a lower extent and generally increased with age and concomitant disease progression (Supplemental Figure 1E). Moreover, the thymic mass was elevated in STAT5B^N642H^ mice compared with that in WT littermates (Figure 1F and Supplemental Figure 1F). Maturation of T cells is accompanied by migration from the thymic cortex to the medulla (37). H&E staining confirmed an increase in the medulla/cortex ratio in STAT5B^N642H^ thymi, in line with the expansion of SP T cells (Figure 1, G and H). Next, we isolated CD8^+^ T cells from STAT5B^N642H^ mice and transplanted them into WT Ly5.1^+^ recipients. Two and 8 weeks after transplant, Ly5.2^+^ CD8^+^ T cells were present in high numbers in spleen, lymph nodes (LNs), bone marrow, blood, liver, or lung but were lacking in thymus, confirming that changes in thymocyte ratios were thymus intrinsic and not caused by infiltration of mature CD8^+^ T cells (Supplemental Figure 1G).

Surprisingly, the *Stat5a^S710F^* GOF mutation’s effect on T cell development was milder compared with STAT5B^N642H^ mice. The *Stat5a^S710F^* mice were analyzed at 28 weeks of age, but the long range of disease latency between 25 and 45 weeks impeded a direct comparison to STAT5B^N642H^ mice, with a consistent end-stage disease around 8–9 weeks of age (Supplemental Figure 1H) (17). These differences highlight the effect of hyperactive STAT5 on T cell development, which consists of expanded immature DN1 thymocytes and strongly increased SP8 thymocyte populations, possibly culminating in T-ALL phenotypes as a result of increased positive selection associated with TCR signaling.

### Hyperactive STAT5A or STAT5B drive activated T cell phenotypes in developing thymocytes.

To understand thymic transcriptional reprogramming by hyperactive STAT5A and STAT5B, we collected DN, DP, and SP8 cells of diseased STAT5B^N642H^ and Stat5a^S710F^ and WT thymi by FACS sorting and performed RNA-Seq (Supplemental Figure 2, A and B). In WT DP thymocytes, *Stat5b* expression was much higher than that of other Stat mRNA, including *Stat5a*. (Supplemental Figure 2, C and D). The majority of differentially expressed genes in STAT5B^N642H^ versus WT thymocytes were upregulated, emphasizing the strong transcriptional activity of hyperactive STAT5B^N642H^ (Supplemental Figure 3, A and B). Mutually upregulated genes by STAT5B^N642H^ across all 3 T cell subsets were enriched for GO terms representing T cell differentiation and activation as well as chemotaxis, interferon type I/II signaling, or host defense (Supplemental Figure 3C). We found that mRNAs encoding for key transcription factors controlling CD8^+^ T cell maturation — such as RUNX3, EOMES, T-bet, and cytotoxic effector molecules granzyme B, perforin, and Fas ligand — were highly upregulated throughout all T cell developmental stages in STAT5B^N642H^ thymi (Figure 1I). Moreover, differentially expressed genes (STAT5B^N642H^ vs. WT in DN, DP, and SP8 subsets) correlated more with signatures of activated than resting T cells (Supplemental Table 1). Differential expression patterns were conserved in sorted DN, DP, and SP8 thymocytes from Stat5a^S710F^ mice (Supplemental Figure 4, A–C). Consistent with strong SP8 lineage commitment caused by hyperactive STAT5, purified STAT5B^N642H^ DP cells gave rise to SP8 lymphomas after transplantation into WT mice (Supplemental Figure 5, A and B).

Normal WT T cells display an activated state mainly in postthymic, mature stages (43). Our RNA-Seq profiles suggested that *STAT5A* and *STAT5B* GOF mutations induce the machinery for high-level activation in immature, unselected T cells, possibly inducing transformation processes through upregulating T cell activation signatures. Importantly, in all analyzed thymic subsets, STAT5B^N642H^ and STAT5A^S710F^ induced transcriptional signatures correlating with human early T cell progenitor–ALL (ETP-ALL) with a reportedly high JAK/STAT activation (44) (Supplemental Figure 5D).

Altogether, these results confirm that STAT5B^N642H^ enhances thymocyte CD8^+^ lineage commitment/expansion. We conclude that STAT5 GOF variants actively contribute to an activated T cell phenotype and a T-ALL–like transcriptome, independent of the T cell development stage and TCR-mediated antigen signaling.

### Hyperactive STAT5 oncoproteins induce thymic T cell tumors upon arrest of T cell development.

**Figure 2:**
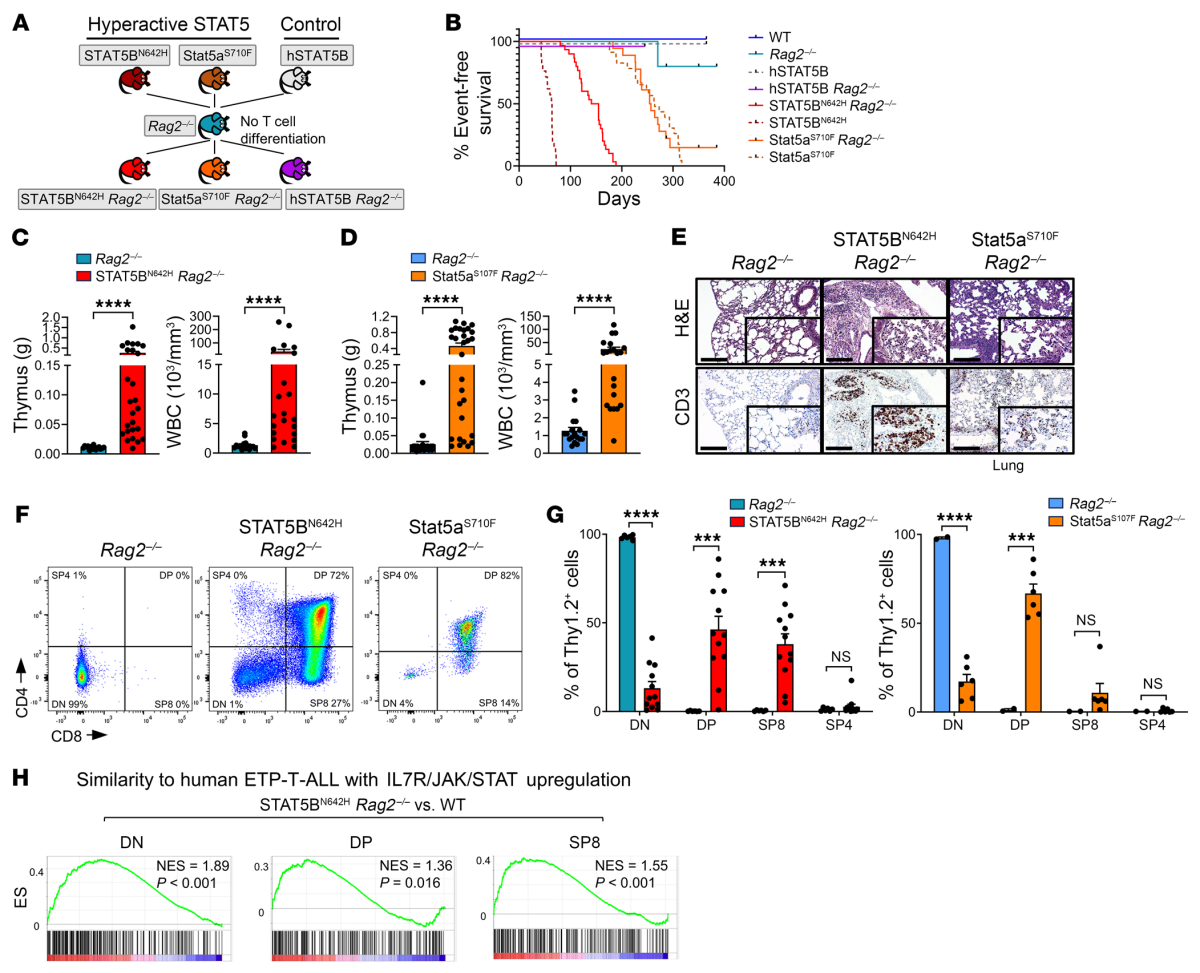
STAT5B^N642H^ induces immature thymic T cell neoplasia in a *Rag2*^–/–^ background. (**A**) Crossing scheme of different STAT5B or Stat5a mutant or WT mice with *Rag2*^–/–^ mice. (**B**) Kaplan-Meier event-free survival plot of all mouse strains of the genotypes resulting from the crossings in **A**. (**C**) Thymus weights and WBC of STAT5B^N642H^ *Rag2*^–/–^ mice (*n* = 26 and 22) compared with *Rag2*^–/–^ littermates (*n* = 15 and 16) and (**D**) Stat5a^S710F^ *Rag2*^–/–^ mice (*n* = 29 and 21) compared with *Rag2*^–/–^ littermates (*n* = 25 and 18). (**E**) H&E and immunohistochemical anti-CD3 staining of lung tissues of STAT5 GOF *Rag2*^–/–^ and *Rag2*^–/–^ mice. Representative of 4 biological replicates. Original magnification: ×10; ×20 (insets); scale bar: 200 μm. (**F** and **G**) Flow cytometry analysis and relative abundance of DN, DP, SP8, and SP4 cells by CD4/CD8 staining gated on Thy1.2^+^ cells in STAT5B^N642H^ *Rag2*^–/–^ (*n* = 12) and *Rag2*^–/–^ (*n* = 6) littermates and Stat5a^S710F^ *Rag2*^–/–^ (*n* = 2) and *Rag2*^–/–^ (*n* = 6) littermates. (**H**) GSEA comparing top 250 upregulated genes in human ETP-ALL (10) to deregulated genes in STAT5B^N642H^ *Rag2*^–/–^ DN or SP8 versus respective WT populations, NES, normalized enrichment score. In **C**, **D**, and **G**, significant differences are indicated as ***P < 0.001, ****P < 0.0001 by a Mann-Whitney *U* test. Data are shown as the mean ± SEM.

Blocking immature stages of T cell development and acquisition of self-renewal capacity through driver oncogenes is a hallmark of T-ALL (7). To prevent mature T cell differentiation and to assess the ability of STAT5 GOF to induce immature, thymic T cell leukemia, we crossed our hyperactive STAT5 mice and STAT5B WT controls with *Rag2*-deficient mice, in which T cell development is blocked at the DN3 stage due to a lack of VDJ recombination and surface TCR αβ/γδ chain expressions (45) (Figure 2A). Notably, in this setting, we did not observe myeloid transformation and only 1 mouse of 30 developed a CD19^+^ B cell lymphoma that was excluded from subsequent analysis (Supplemental Figure 6A). All other STAT5 GOF *Rag2*^–/–^ mice developed a dominant T-ALL–like phenotype, irrespective of sex. STAT5B^N642H^ *Rag2*^–/–^ mice developed terminal disease between 15 and 22 weeks of age, representing a substantially longer latency than in STAT5B^N642H^ mice (Figure 2B). In comparison, Stat5a^S710F^ *Rag2*^–/–^ mice succumbed to disease significantly (*P* < 0.0001) later (25–45 weeks) than STAT5B^N642H^ *Rag2*^–/–^ mice, similar to Stat5a^S710F^ mice. The disease in STAT5 GOF *Rag2*^–/–^ mice was characterized by a massive increase in thymus/spleen weights and WBC counts compared with *Rag2*^–/–^ littermates, where thymus enlargement was most drastic, also compared with WT and STAT5B^N642H^ mice (Figure 2, C and D, and Supplemental Figure 6, B–D). An increase in the numbers of Thy1.2-expressing cells by flow cytometry and high CD3 expression assessed by immunohistochemistry indicated strong thymic T cell expansion in STAT5 GOF *Rag2*^–/–^ mice, despite the loss of VDJ recombination (Supplemental Figure 6, E and F), coupled with T cell infiltration into the lung (Figure 2E). In contrast, human WT STAT5B (hSTAT5B) *Rag2*^–/–^ mice did not show any sign of disease when sacrificed at 35 weeks of age, similar to *Rag2*^–/–^ and WT control mice (Figure 2B and Supplemental Figure 6G).

Next, we aimed to evaluate T cell immunophenotypic surface marker expression in the thymus at terminal disease by flow cytometry. In line with previous studies, staining for CD4 and CD8 was absent in *Rag2*^–/–^ thymi. Strikingly, in all STAT5B^N642H^ *Rag2*^–/–^ and Stat5a^S710F^ *Rag2*^–/–^ mice, the majority of thymocytes was constituted by DP and SP8 cells, indicating that STAT5 hyperactivation overcomes the developmental block in the DN stage of *Rag2*^–/–^ thymocytes and induces expression of CD4 and CD8 (Figure 2, F and G). Thymocytes of hSTAT5B *Rag2*^–/–^ mice revealed no significant upregulation of CD4 or CD8 compared with *Rag2*^–/–^ littermates (Supplemental Figure 6H). We also detected elevated thymus weights and expression of CD4 and CD8 in younger (10- to 14-week-old), nonterminally diseased STAT5B^N642H^ *Rag2*^–/–^ mice (Supplemental Figure 6, I and J).

Next, we performed RNA-Seq on FACS-sorted thymic DN cells of *Rag2*^–/–^ mice and DN, DP, and SP8 cells of diseased STAT5B^N642H^ *Rag2*^–/–^ and Stat5a^S710F^ *Rag2*^–/–^ mice (Supplemental Figure 7A). Global changes in gene expression appeared similar in STAT5B^N642H^ *Rag2*^–/–^ and Stat5a^S710F^ *Rag2*^–/–^ thymocytes compared with *Rag2*^–/–^ thymocytes, again indicating overlapping functions of both hyperactive *STAT5A* and *STAT5B* gene variants (Supplemental Figure 7, B and C) in their capacity to transform immature T cells. Importantly, the STAT5B^N642H^-driven thymic neoplasms transcriptionally correlated with human ETP-ALL (Figure 2H), whereas this correlation was weaker for STAT5A^S710F^-driven thymic disease, except for DN cells (Supplemental Figure 8A). Altogether, these results confirm that STAT5A^S710F^ and the clinically more relevant STAT5B^N642H^ are drivers for T-ALL, suggesting their targeting in T-ALL.

### Immature thymocytes of STAT5B^N642H^ Rag2^–/–^ mice transform to T-ALL.

The increase in thymus size of diseased STAT5B^N642H^ *Rag2*^–/–^ mice was the most drastic difference compared with *Rag2*^–/–^, WT, or STAT5B^N642H^ mice (Supplemental Figure 5D), suggesting the development of immature T cell neoplasia. While cortical and medullary regions were histologically distinguishable in WT and STAT5B^N642H^ thymi, only cortical regions were identified in *Rag2*^–/–^ or STAT5B^N642H^ *Rag2*^–/–^ thymi (Supplemental Figure 8B). We found widespread, strong expression of the proliferation marker Ki67 (Supplemental Figure 8C) and the clinically relevant T-ALL marker terminal deoxynucleotide transferase (TdT) in STAT5B^N642H^ *Rag2*^–/–^ thymi (Supplemental Figure 8D). High levels of TdT indicated immature thymocyte expansion and a phenotype similar to ETP-ALL. This supports the above findings that STAT5B^N642H^ *Rag2*^–/–^ thymocytes remain immature.

Comparing RNA-Seq data from 4 mouse genotypes (WT, STAT5B^N642H^, *Rag2*^–/–^, and STAT5B^N642H^ *Rag2*^–/–^), we found elevated levels of *Pim1* exclusively in STAT5B^N642H^ *Rag2*^–/–^ thymocytes (Supplemental Figure 9A), as confirmed by Western blot (Supplemental Figure 9B). PIM1 kinase is a well-described oncogenic, direct STAT5-target gene that is targeted in T-ALL (46, 47). Our findings suggest that STAT5 GOF mutations promote immature T-ALL initiation and progression.

### STAT5B^N642H^ upregulates TCR pathway genes.

**Figure 3:**
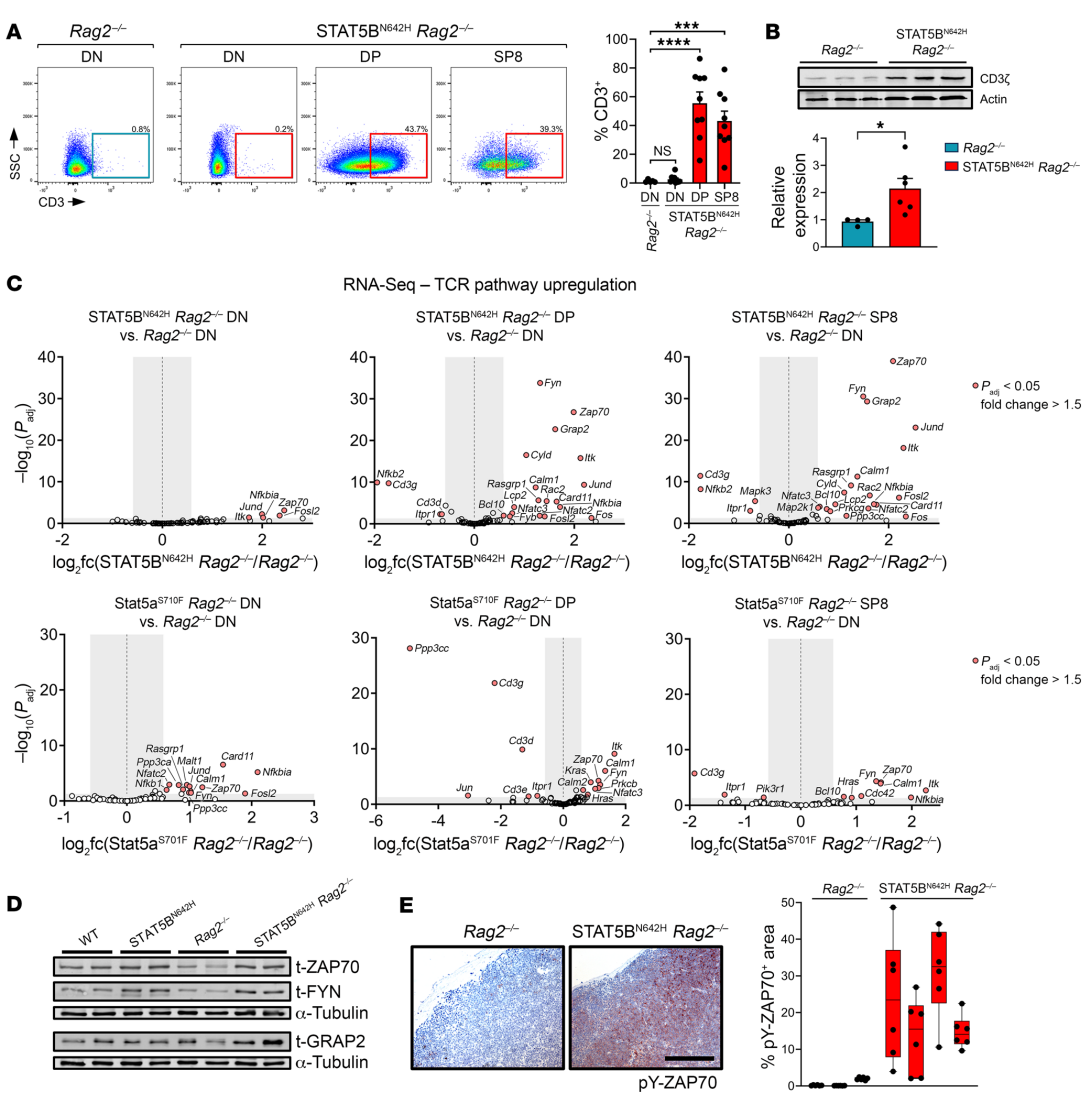
STAT5B^N642H^ *Rag2*^–/–^ T-ALL cells upregulate CD3 surface expression and TCR pathway genes. (**A**) Flow cytometry analysis for CD3 surface expression in DN, DP, or SP8 cells of thymi from STAT5B^N642H^ *Rag2*^–/–^ (*n* = 9) or *Rag2*^–/–^ (*n* = 5) littermates. (**B**) Western blot analysis for CD3ζ expression in protein extracts of whole thymi of STAT5B^N642H^ *Rag2*^–/–^ or *Rag2*^–/–^ mice and quantification thereof, with *n* = 6 STAT5B^N642H^ *Rag2*^–/–^and *n* = 4 *Rag2*^–/–^. (**C**) Volcano-plots of RNA-Seq data of DN, DP, and SP8 (STAT5B^N642H^ *Rag2*^–/–^ and Stat5a^S710F^ *Rag2*^–/–^) versus DN (respective *Rag2*^–/–^ littermates) cells; *P*_adj_ and logfc determined by DESeq analysis. (**D**) Western blot analysis for total (t) ZAP70, t-FYN, and t-GRAP2 expression in protein extracts of whole thymi of indicated mouse genotypes. (**E**) Representative immunohistochemical pY-ZAP70 staining of thymi of STAT5B^N642H^ *Rag2*^–/–^ (*n* = 4) or *Rag2*^–/–^ (*n* = 3) mice and quantification thereof; 6 distinct areas per slide were subjected to analysis using ImageJ (NIH) and the Colour Deconvolution2 plugin. Original magnification, ×20; scale bar: 200 μm. In **A** and **B**, significant differences are indicated as *P < 0.05, ***P < 0.001, ****P < 0.0001 by 1-way ANOVA with Dunnett’s multiple comparison test (**A**) or unpaired 2-tailed Student’s *t* test (**B**). Data are shown as the mean ± SEM.

Next, we addressed the molecular basis of immature T cell neoplasm development. The absence of surface CD3 (sCD3) attenuates the transition from DN to DP T cells, whereas CD3 stimulation or transgenic TCR expression induces a transition to the DP stage in RAG-deficient mice (48). DN cells were sCD3-negative as expected, but surprisingly, DP and SP8 cells from STAT5B^N642H^ *Rag2*^–/–^ thymi expressed elevated levels of sCD3, as a possible prerequisite for this TCR-driven progress in thymic development (Figure 3A). Notably, the CD3ζ chain was expressed at higher levels in STAT5B^N642H^ *Rag2*^–/–^ mice than in *Rag2*^–/–^ thymi (Figure 3B).

Previous studies suggest that transition to the DP stage despite recombination deficiency can result from signaling downstream of the TCR pathway (48). Based on our above finding, we therefore more closely evaluated the transcription of genes involved in the TCR signaling cascade. Strikingly, STAT5B^N642H^ induced significant upregulation of 18 (DP) and 20 (SP8) genes of 71 genes encoding proteins that are implicated in TCR signaling (Figure 3C). Fewer of these genes were significantly deregulated in STAT5B^N642H^ *Rag2*^–/–^ DN cells, in line with weaker sCD3 expression in this subset (Figure 3C). GSEA confirmed these results, in which gene expression profiles of DP and SP8 cells correlated more with TCR pathway genes than DN cells (Supplemental Figure 9C). Surprisingly, this signature was less pronounced in thymi of Stat5a^S710F^ *Rag2*^–/–^ mice, in which only DN cells displayed moderate upregulation of the same genes (Figure 3C). We confirmed that STAT5B^N642H^ *Rag2*^–/–^ thymocytes expressed higher levels of ZAP70 and FYN tyrosine kinases and the scaffold kinase adaptor GRAP2 protein (Figure 3D and Supplemental Figure 9D) and found higher levels of phosphorylated ZAP70 in the thymi of STAT5B^N642H^ *Rag2*^–/–^ mice by immunohistochemistry (Figure 3E). Moreover, *Ago2* transcription was upregulated in the thymic subsets that also displayed upregulation of TCR pathway genes (Supplemental Figure 9E). While AGO2 is a regulator of miRNA processing, it has been recently attributed to noncanonical functions in TCR signaling upregulation by complex formation with, for example, ZAP70 (49). We conclude that to mimic TCR signaling hyperactive STAT5 upregulates expression of genes encoding, for example, CD3ζ, AGO2, and GRAP2 or kinases like ZAP70, FYN, and ITK.

### Human T-ALL expresses high levels of TCR pathway genes.

**Figure 4:**
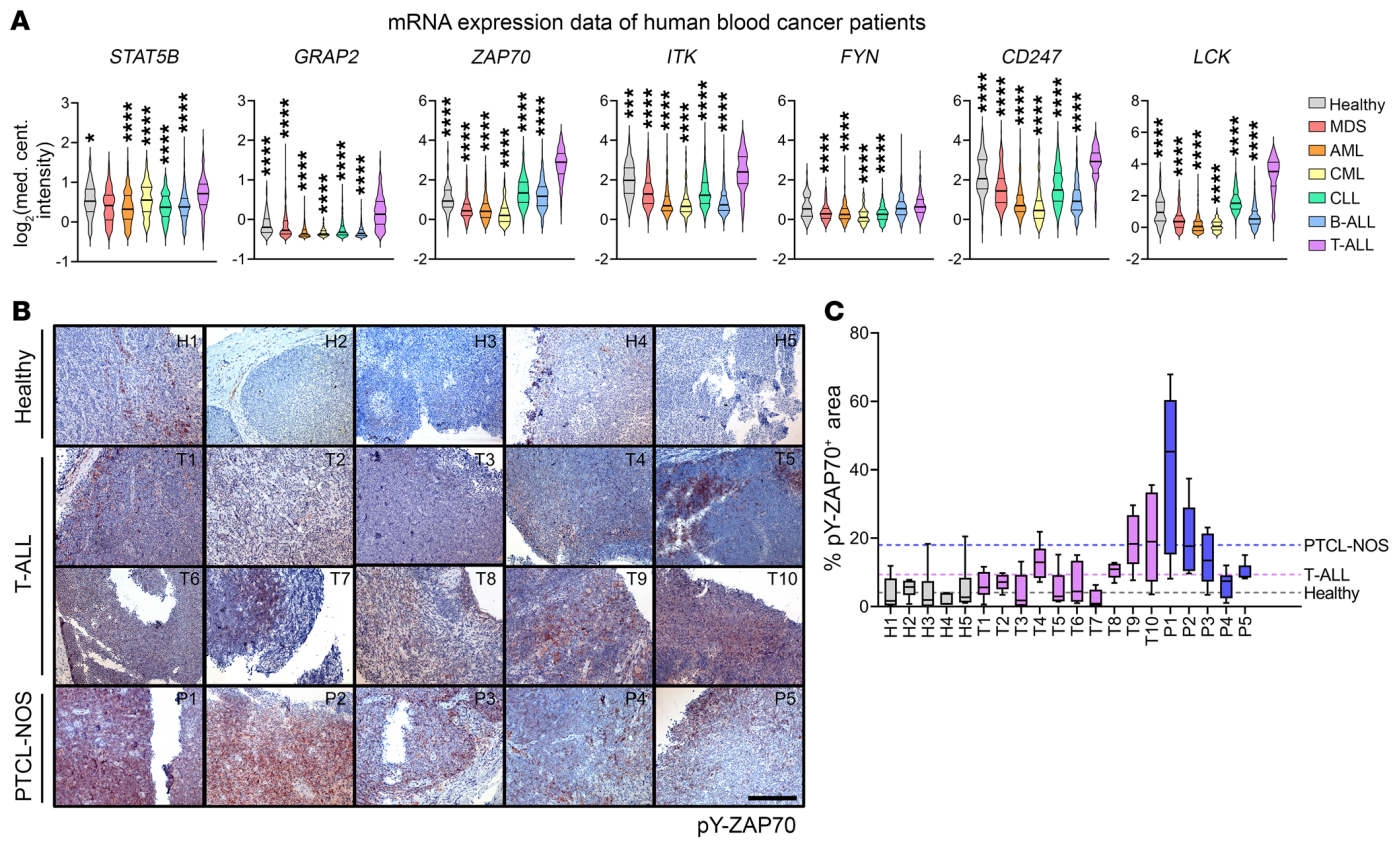
T cell kinases are upregulated and active in human T-ALL. (**A**) *STAT5B*, *GRAP2*, *ZAP70*, *ITK*, *FYN*, *CD247*, and *LCK* mRNA expression data of patients with from different blood cancers and healthy bone marrow control cells. Data extracted from the “Haferlach Leukemia” study from the Oncomine database. MDS, myelodysplastic syndrome (*n* = 206); AML, acute myeloid leukemia (*n* = 542); CML, chronic myelogenous leukemia (*n* = 76); CLL, chronic lymphocytic leukemia (*n* = 448); B-ALL, B cell acute lymphoblastic leukemia (*n* = 576); T-ALL, T cell acute lymphoblastic leukemia (*n* = 174). (**B**) Representative immunohistochemical staining of lymph nodes from healthy donors (*n* = 5) and tumor samples from patients with T-ALL (*n* = 10) and patients with from peripheral T cell lymphoma (*n* = 5), not otherwise specified (PTCL-NOS), for pY-ZAP70. Original magnification: ×20; scale bar: 200 μm. (**C**) Quantification of areas positive for pY-ZAP70 in patient samples shown in **B**; 6 distinct areas per slide were subjected to analysis using ImageJ (NIH) and the Colour Deconvolution2 plugin. In **A**, significant differences are shown for comparisons of each data set to T-ALL and are indicated as *P < 0.05, ***P < 0.001, ****P < 0.0001 by 1-way ANOVA with Dunnett’s multiple comparison test. (**A** and **C**) Data are depicted as violin and box-and-whiskers plots, respectively, showing median, 25th and 75th quartiles, ranging from minimum to maximum values.

Next, we explored gene expression profiles of patients with T-ALL to evaluate expression levels of TCR pathway genes. In 3 independent studies, *ZAP70*, *FYN*, *GRAP2*, *ITK*, and *CD247* (encoding CD3ζ) were highly upregulated in patients with T-ALL compared with individuals acting as healthy controls or people with other hematopoietic cancers, similar to our murine study (Figure 4A and Supplemental Figure 10A). Additionally, *LCK*, another key regulator of T cell activation, was highly upregulated in these data sets (Figure 4A), although we did not observe significant *Lck* upregulation in our 4 neoplastic mouse models. Furthermore, we found that expression levels of *ZAP70*, *FYN*, *GRAP2*, *ITK*, and *LCK* as well as two further TCR pathway kinases, *PLCG1* and *LAT*, were highest in human T-ALL cell lines compared with other hematopoietic cancer lines (Supplemental Figure 10B). Oncogenic TCR signaling has already been reported in PTCL (50–53), and higher expression levels of TCR pathway kinases in T-ALL compared with PTCL strengthen the hypothesis that they are key players in immature T cell transformation. However, continuously high expression of these genes in PTCL confirmed that TCR signaling might be targetable in a broad range of T cell cancers. Examining publicly available CRISPR/Cas9 screening data, we found that in T-ALL cell lines the dependency scores of *LCK*, *ZAP70*, and *CD247* were higher than for other TCR pathway genes, reflecting that the CD3ζ-ZAP70 interaction could be a vulnerable node in T-ALL (Supplemental Figure 10C). Moreover, high ZAP70 expression correlated with a higher risk of T-ALL relapse in patients (Supplemental Figure 10D). We analyzed whole-exome sequencing in conjunction with RNA-Seq data of human T-ALL subtypes and found that immature T-ALL cells with *JAK/STAT* GOF mutations had increased *ZAP70* expression levels compared with unmutated ones (Supplemental Figure 10E). Immunostaining of individual biopsies from patients with T-ALL revealed higher average ZAP70 activation than in LNs from healthy individuals, which further supports the concept of an activated T cell signature in a subfraction of T-ALL cells in patient biopsies. However, pY-ZAP70 was lower in T-ALL samples than in PTCL not otherwise specified (PTCL-NOS) samples (Figure 4, B and C). Thus, our data indicate that active STAT5 or CD3ζ-ZAP70 signaling could be relevant targets in human T-ALL and, possibly also, in several types of PTCL, in which STAT5B^N642H^ is also found (19).

### Human T-ALL cell lines with active STAT5 respond to pharmacologic inhibition of STAT3/5 or ZAP70.

**Figure 5:**
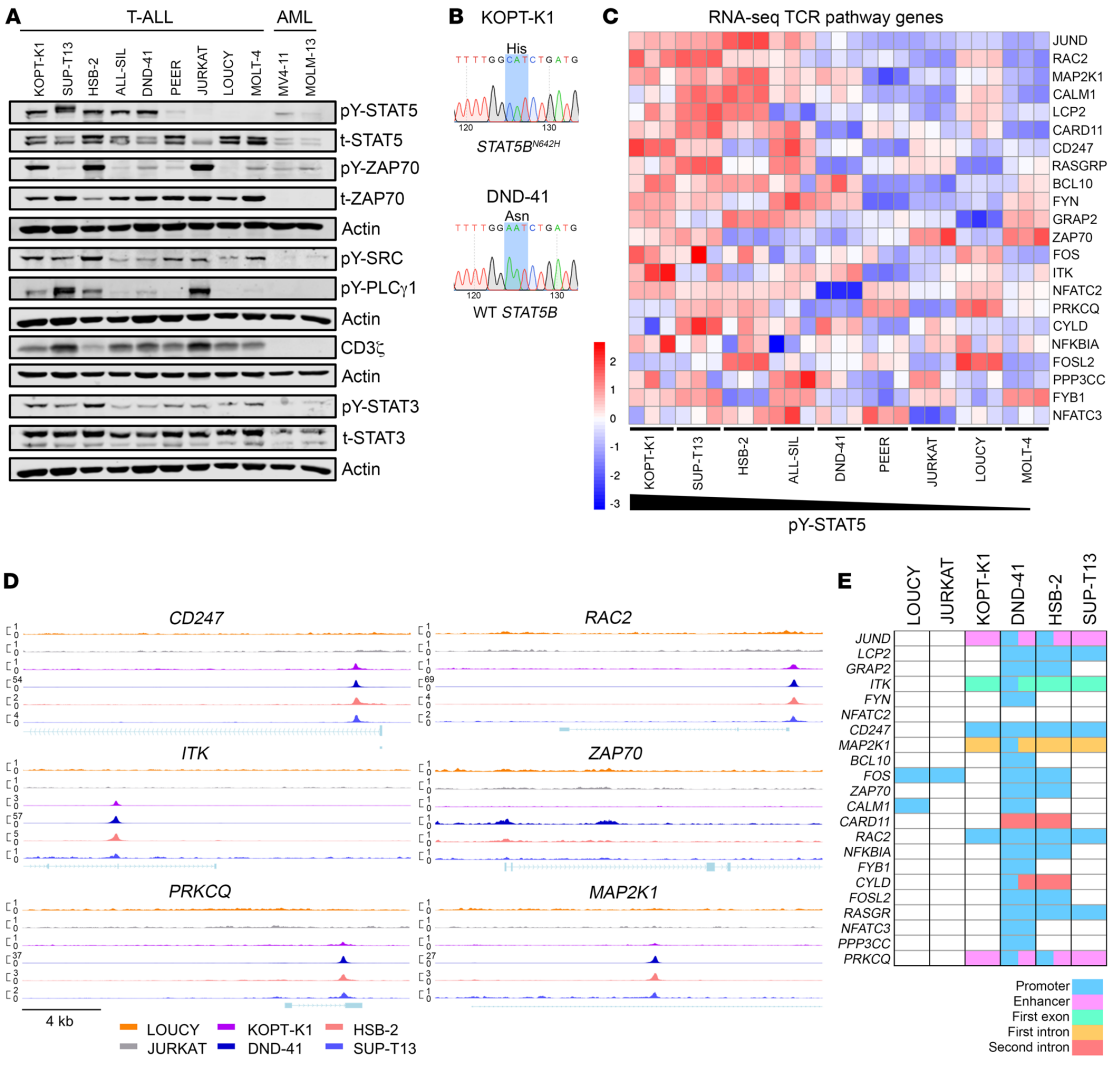
Human T-ALL cell lines with high STAT5 activation display upregulated expression of TCR pathway genes. (**A**) Basal levels of phosphorylated (pY) and total (t) STAT5, STAT3, and TCR pathway proteins in 9 T-ALL and 2 AML control cell lines, evaluated by Western blot analysis. Actin served as loading control. (**B**) Sanger sequencing of the SH2 and TAD domains of STAT5B. cDNA was obtained from isolated RNA of KOPT-K1 and DND-41 cells. The codon for amino acid position 642 is highlighted in blue. (**C**) *Z* scores of raw counts of TCR pathway genes from RNA-Seq data from 9 T-ALL cell lines. Three biological triplicates were acquired from each cell line and cell lines were arranged according to their pY-STAT5 level as determined by Western blot. (**D**) ChIP-Seq peak signals indicating STAT5B binding at the promoter region of 6 TCR pathway genes as indicated in 6 T-ALL cell lines. Normalized ChIP-Seq signal (CPM) is indicated on the *y* axis. (**E**) Peak analysis for STAT5B binding sites in TCR pathway genes from 6 T-ALL cell lines and indication of peak localization within respective genes or enhancers.

To assess the potential of targeting STAT5 and downstream kinases in T-ALL, we profiled 9 human T-ALL cell lines for their STAT5 and TCR signaling activity. Five T-ALL cell lines (KOPT-K1, SUP-T13, HSB-2, ALL-SIL, and DND-41) displayed high STAT5 activation by Western blotting. STAT5 activation was lower in the PEER cell line and the lowest in JURKAT, LOUCY, and MOLT-4 cells, similar to the AML lines MOLM-13 and MV4-11 (Figure 5A). Sanger sequencing on mRNA encoding the SH2 domain of STAT5B identified a potentially novel biallelic N642H-driver mutation in KOPT-K1 cells (Figure 5B). This explains the high level of STAT5 activation observed in this cell line and validates it as a relevant human model to study STAT5B^N642H^ in T-ALL. The DND-41 cell line lacked *STAT5B* mutations (Figure 5B) but is known to carry activating *IL7R* (*p.L242_L243insLSRC*) and loss-of-function *PTPRC* (W764*) mutations, leading to active IL-7Rα/JAK1/STAT5B signaling (8) (Figure 5A). None of the other tested T-ALL cell lines harbored GOF SH2 domain mutations in *STAT5B* or in the JH2 domain of *JAK1* or *JAK3* (further targeted Sanger sequencing data not shown).

Next, we performed RNA-Seq of T-ALL cell lines to further evaluate the effect of STAT5 activity on gene transcription of TCR pathway members. We found higher expression of these genes in cell lines with higher STAT5 activation (Figure 5C). In line with this, we found higher CD3ζ chain and ZAP70 protein expression, with ZAP70 and downstream SRC and PLCγ1 activation in T-ALL cell lines confirming active kinase signaling downstream of the TCR in T-ALL (Figure 5A). Levels of active and total STAT3 were more stable between the T-ALL and reduced compared with the AML cell lines (Figure 5A).

To further elucidate whether STAT5 is directly involved in TCR pathway gene regulation, we performed ChIP-Seq analysis for STAT5B in 4 T-ALL cell lines with high pY-STAT5 levels (KOPT-K1, DND-41, HSB-2, and SUP-T13) and 2 with low pY-STAT5 levels (LOUCY and JURKAT; Figure 5A). The robustness of our ChIP-Seq analysis was confirmed by STAT5B peaks in the bona fide STAT5 target genes *BCL2*, *BCL2L1*, *SOCS3*, and *RUNX3* in KOPT-K1, DND-41, HSB-2, and SUP-T13 cells in (Supplemental Figure 11A). Furthermore, we found that STAT5 binds at promoters, enhancers, introns, or exons of TCR pathway genes, including *CD247* and downstream effector molecules *ZAP70*, *ITK*, *MAP2K1*, *PRKCQ*, *RAC2* and others (Figure 5, D and E), specifically in the cell lines with high STAT5 activity. This indicates that these genes could be transcriptional targets upregulated by active STAT5 in particular.

**Figure 6:**
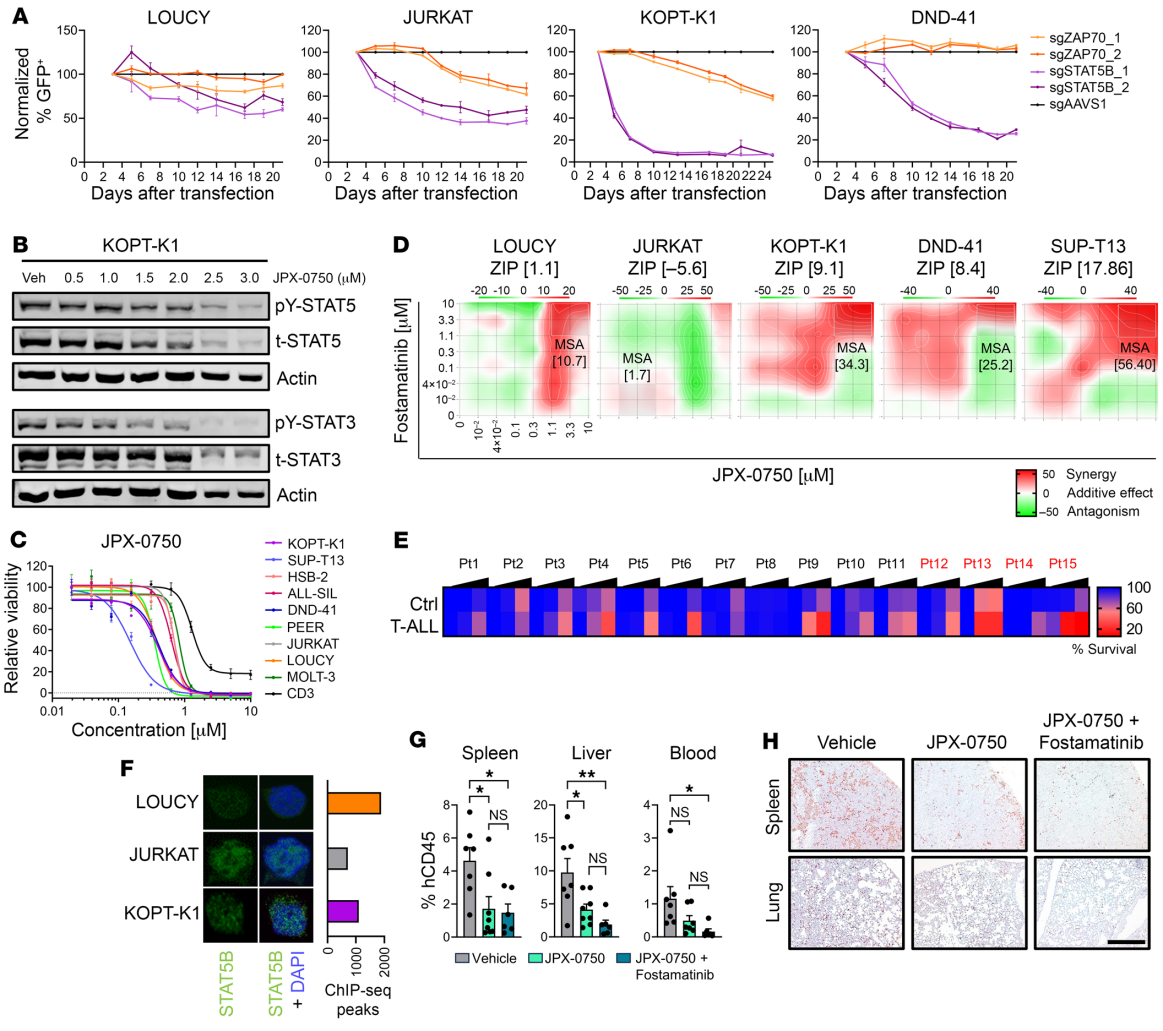
T-ALL cells with elevated TCR pathway gene expression and STAT5 activation respond to ZAP70 and STAT5 inhibition. (**A**) Growth of T-ALL cell lines upon *STAT5B* or *ZAP70* CRISPR/Cas9 knockout via GFP tracing, normalized to day 3 after infection, and *AAVS1* and *RPL17* as control genes. (**B**) Western blot analysis for pY-STAT5/-STAT3 and t-STAT5/-STAT3 in KOPT-K1 cells treated 24 hours with JPX-0750 at indicated concentrations or vehicle (Veh), with actin as loading control. (**C**) Dose-response curve for T-ALL cell lines or human nonleukemic CD3 cells treated with JPX-0750. Three or more independent experiments in technical triplicates were performed. (**D**) Dose-response synergy analysis of the indicated 2-drug combination in T-ALL cells after 72 hours treatment. The most synergistic area (MSA) is highlighted (white rectangle) with the respective MSA score. One representative plot of 2 independent experiments is shown. (**E**) Percentage survival of nonleukemic control and T-ALL cells from primary patient samples treated for 24 hours with JPX-0750 at 100, 1,000, or 10,000 nM. Mean values of respective concentration versus DMSO control in 2 technical replicates are shown. Relapsed patients are marked in red. (**F**) Confocal microscopy of LOUCY, JURKAT, and KOPT-K1 T-ALL cells stained for STAT5B (green) and DAPI (blue), and the number of peaks found in STAT5B ChIP-Seq of respective cell lines. (**G**) Fractions of hCD45^+^ KOPT-K1 cells in spleen, liver, and blood after transplantation into NSG mice and treatment with JPX-0750 (*n* = 8) alone or in combination with fostamatinib (*n* = 6) or vehicle (*n* = 7) for 21 days. (**H**) Representative immunohistochemical staining for CD3 in the spleens and lungs of these mice. Original magnification: ×10; scale bar: 400 μm. In **G**, significant differences are indicated as *P < 0.05, **P < 0.01 by 1-way ANOVA with Tukey’s multiple comparison test. In **A**, **C**, and **G**, data are shown as the mean ± SEM.

Next, we performed CRISPR/Cas9-mediated knockout of *STAT5B* and *ZAP70* in 4 selected cell lines. *STAT5B* knockout induced rapid depletion of KOPT-K1, DND-41, JURKAT, and, to a lesser extent, LOUCY cells (Figure 6A). *ZAP70* knockout depleted KOPT-K1 and JURKAT cells after longer cultivation, while DND-41 and LOUCY cells were not significantly affected (Figure 6A).

To test pharmacologic inhibition of these proteins, we used JPX-0750 (54, 55), a small-molecule STAT3/5 degrader, which prevents compensatory STAT3 upregulation upon STAT5 inhibition as an escape route, or the established SYK inhibitors fostamatinib or gusacitinib, since ZAP70 is 1 of 2 members of the SYK protein family. Gusacitinib is a dual SYK/JAK inhibitor for which the FDA has granted fast track designation for chronic hand eczema (56), also targeting JAKs. Fostamatinib is an FDA-approved SYK inhibitor for chronic immune thrombocytopenia (57) that also targets FLT3, LCK, and LYN. Treatment of T-ALL cells with JPX-0750 rapidly abrogated STAT5 and STAT3 but also ZAP70 activation and expression, suggesting direct dependency on STAT3/5. JPX-0750 treatment induced diminished levels of PIM1, but BCL-2 expression remained normal (Figure 6B and Supplemental Figure 11, B and C). While *PIM1* and *BCL2* are STAT5-target genes, BCL-2 is also independently regulated by the PI3K/AKT/mTOR pathway, which is in turn interwoven with STAT5 signaling in T cell metabolism (47, 58). Remarkably, T-ALL cells were more sensitive to JPX-0750 compared with sorted CD3^+^ T cells from healthy donors (Figure 6C). Moreover, we found that T-ALL cells with high pY-STAT5 levels and downstream TCR kinase activation responded to fostamatinib or gusacitinib treatment. Fostamatinib displayed a higher selectivity to those cell lines with higher kinase activation or ZAP70 expression (KOPT-K1, HSB-2, JURKAT, ALL-SIL, DND-41, SUP-T13, MOLT-4) with higher sensitivity compared with CD3^+^ control cells (Supplemental Figure 11D). Importantly, T-ALL cells treated with these selective kinase blockers displayed reduced levels of ZAP70 and STAT5 activation without significantly altering their total protein levels (Supplemental Figure 11E). Treatment of *ZAP70*-knockout cells with fostamatinib resulted in a 38% increase of the IC_50_ dose compared with control cells (Supplemental Figure 11F). This suggests that fostamatinib effectively targets ZAP70 but also that other kinases such as FLT3, SYK, LCK, or LYN might be affected. JAK/STAT and TCR signaling are distinct pathways, and our findings suggest cooperative cross-regulation. Thus, we tested whether combined inhibition leads to synergy. Notably, fostamatinib synergized with JPX-0750 selectively in KOPT-K1, DND-41, and SUP-T13 cells with high pY-STAT5 and elevated TCR pathway gene expression, while no significant synergy was observed in the control LOUCY and JURKAT cells (Figure 6D). Hence, our data suggest that combinatorial treatment with SYK and STAT5 inhibitors appears promising for patients with T-ALL with activated STAT5/ZAP70. Next, we treated primary human T-ALL cells (Supplemental Table 2) with JPX-0750 or fostamatinib and found higher sensitivity to JPX-0750 in T-ALL cells compared with nontumor cells in 12 of 15 samples (Figure 6E). Fostamatinib selectively killed T-ALL cells in 2 samples; importantly, the sample that responded best to JPX-0750 (patient 10) was also highly sensitive to fostamatinib (Supplemental Figure 11G). Remarkably, both fostamatinib-responders were relapsed patients with T-ALL, indicating that more progressive and aggressive disease stages might be more sensitive to ZAP70 inhibition (Supplemental Table 2). To further assess why T-ALL cells with low STAT5 activity responded to the targeted STAT3/5 degradation, we performed immunofluorescence microscopy of JURKAT and LOUCY cells and detected nuclear STAT5 in both cell lines, with KOPT-K1 serving as a positive control. We specifically detected both STAT5A and STAT5B variants in LOUCY cells and only STAT5B in JURKAT cells (Figure 6E and Supplemental Figure 12A), which lack STAT5A expression (Figure 5A). In line with this, the total number of genes that were bound by STAT5B in our ChIP-Seq analysis was comparable among LOUCY, JURKAT, or KOPT-K1 cells (Figure 6F). Finally, we used JPX-0750 and fostamatinib to confirm their efficacy in vivo. Strikingly, the number of intravenously transplanted KOPT-K1 cells in the spleens, liver, and blood was reduced in mice treated with JPX-0750 or JPX-0750 and fostamatinib. Of note, only the combination treatment led to a significant reduction of T-ALL cells in the blood (Figure 6, G and H). Transplanted DND-41 cells were detected in the bone marrow but did not sufficiently engraft in spleen, liver, or blood. Nevertheless, JPX-0750 treatment reduced DND-41 cell fractions in the bone marrow, without reaching statistical significance (Supplemental Figure 12B). Altogether, we conclude that mutated STAT5A and STAT5B are oncoproteins in T-ALL. Their hyperactivation drives upregulation of genes of the TCR signaling axis, representing future vulnerable signaling nodes for targeted therapy.

## Discussion

We genetically prove that hyperactive STAT5A or STAT5B change thymic T cell development and promote leukemic transformation of immature T cells. We show that *STAT5A* and *STAT5B* GOF mutations induce T-ALL, without driver mutations in upstream *JAK1*, *JAK3*, or *IL7R* and define them as oncogenes in T-ALL. To date, diagnostic screening for STAT5 hyperactivation to adjust therapy is not standard procedure for patients with T-ALL. We suggest that hyperactive STAT5 mimics TCR signaling in T-ALL, thereby driving and initiating the disease by directly or indirectly activating transcription of TCR pathway genes. In KOPT-K1 cells isolated from a male child with T-ALL we discovered a biallelic *STAT5B^N642H^* mutation, defining it as suitable patient-derived model to study the most frequent *STAT5B* driver mutation. In contrast to the commonly applied approach of JAK inhibition, we present what we believe to be the first direct STAT5-targeting approach in T-ALL and show that STAT5 and ZAP70 signaling are essential in a subset of human T-ALL. Pharmacologic intervention using STAT3/5 and ZAP70 inhibitors either alone or synergistically blocked human T-ALL cell growth in a xenograft T-ALL model. STAT5B^N642H^ has been found to correlate with a higher risk of relapse (24), and thus, we emphasize that our results are particularly relevant for relapsed or therapy-resistant T-ALL, which represent the major challenges in T-ALL treatment. We predict that, owing to progress in personalized medicine and clinical management of patients with T-ALL, significant numbers of *STAT5B^N642H^* driver mutations will be found that need to be treated with targeted therapy. Owing to the significant rate of STAT5B mutations in 9 different T cell cancers, which exhibit STAT5 activation status and the essential genetic role for STAT5 in T cell differentiation, function, proliferation, and survival, we postulate that our findings in T-ALL extend to the large group of approximately 30 different mature T cell cancers (such as PTCL) (59). This is supported by the fact that activating *STAT5B* mutations and particularly *STAT5B^N642H^* also occur in T cell large granular lymphocytic leukemia, T cell prolymphocytic leukemia, and various γδ T cell–derived leukemias/lymphomas (18). Furthermore, STAT5 activation is targetable in cutaneous T cell lymphomas, bearing *STAT5A* and *STAT5B* copy number gains, and PDGFRβ-driven anaplastic large cell lymphoma (55, 60). STAT5 activating kinase triggers might lead to similar molecular consequences as *STAT5B^N642H^*, highlighting broad applicability of our results. STAT5A and STAT5B have been validated as oncogenic drivers of T cell malignancies in mouse models (16–18). Higher basal expression levels of STAT5B over STAT5A likely contribute to a dominant role of STAT5B in T cell biology and leukemogenesis, and hyperactive STAT5A requires stronger overexpression to transform T cells (17). This might explain the lower effect of STAT5A^S710F^ on DN1–4, DP, and SP4/8 stage composition compared with STAT5B^N642H^. Comprehensive RNA-Seq analysis revealed enhanced T cell activation and lineage commitment with significant RUNX3, EOMES, or T-bet upregulation by STAT5B^N642H^ or STAT5A^S710F^ expression in purified DN, DP, and SP8 thymocytes. Our data share parallels with findings from the O′Shea and Hennighausen groups, demonstrating that murine *Stat5b* knockout had a higher effect on T cell differentiation and function than *Stat5a* knockout, whereas individual *Stat5a* or *Stat5b* knockout was highly similar at the transcriptome level (61). Normally, T cell development and selection are highly sensitive processes, where physiological negative and positive selection lead to elimination of up to 95% of thymocytes (62). Thus, it is surprising that T cells bearing hyperactive STAT5 most likely overcome apoptosis in thymic selection processes in the thymus and give rise to mature postthymic T cells (16).

Studies have identified mutated or overexpressed IL-7R, JAK1, or JAK3 as oncoproteins in patients with T-ALL and validated them as oncogenic drivers in mouse models (8, 9, 12, 14, 63). Many of these studies suggested that IL-7R/JAK1/3 signals via STAT5. The question of whether *STAT5A* or *STAT5B* GOF mutations can directly induce T-ALL remained unanswered. STAT5 GOF alone failed to induce a T-ALL–characteristic arrest in T cell development in the presence of VDJ recombination. To counteract mature T cell generation, we induced arrest in thymic development in STAT5 GOF mice by crossing them with *Rag2*^–/–^ mice. Indeed, these mice succumbed to a T-ALL–like disease, mainly characterized by massive thymic enlargement and dominant thymic TdT expression. *Rag2* deficiency may imitate other common genetic lesions in human T-ALL, such as chromosomal rearrangements (4). Whether STAT5 mutations cooperate with these lesions, e.g., involving *TAL1*, *HOXA1*, or *TLX1*, or with hyperactive *NOTCH1* mutations remains to be explored. We revealed that both STAT5 GOF variants launch a transcriptional program that is transcriptionally similar to ETP-ALL. However, the lack of comprehensive, subtype-specific transcriptomes of patients exacerbates definite conclusions about the T-ALL subtype of our mice. Moreover, STAT5B^N642H^ occurs in several clinical T-ALL subtypes (4, 24), highlighting the genetic heterogeneity of this disease. Most tumor cells in STAT5 GOF *Rag2*^–/–^ mice are DP or SP8, suggesting progression in thymic development beyond the DN stage, despite TCR deficiency due to loss of VDJ rearrangement. This resembles induction of thymocyte differentiation promoted by LCK or NPM-ALK overexpression or CD3 cross-linking in RAG-deficient mice (48, 64). However, comprehensive analyses to better understand the transcriptional regulation of these processes by NGS efforts have not been performed. Our RNA-Seq data provide mechanistic explanations revealing a pronounced transcriptional upregulation of genes encoding kinases or adapter proteins that operate downstream of the TCR pathway by STAT5 activation. Higher STAT5 activation by STAT5B^N642H^ also correlated with higher ZAP70 activation in our mouse model. Whether this is a direct effect of STAT5B^N642H^ or a consequence of upregulation of other TCR signaling components remains unclear.

Translating our findings, we found that high expression of these genes is specific to T-ALL compared with other more mature T cell cancer types in humans. Most interestingly, their expression values are higher in T-ALL than in PTCL, where aberrant TCR signaling and its targeting have been proposed in several disease subtypes (50–53). Our findings in mice are consistent with a transcriptional upregulation of important TCR signaling genes, such as *CD247*, *JUND*, *RAC2*, *MAP2K1*, *CALM1*, *LCP2*, *FYN*, *GRAP2*, and *ZAP70*, correlating with STAT5 activity in human T-ALL cells. However, pY-ZAP70 levels were lower in human T-ALL samples than in PTCL-NOS samples but still elevated compared with LNs from healthy individuals. Moreover, ChIP-Seq analysis revealed that STAT5B selectively binds to the promoter of 21 TCR signaling genes in at least one T-ALL cell line. We found strong activation of TCR signaling proteins ZAP70, SRC, and PLCγ1 in KOPT-K1, HSB-2, and SUP-T13 cells as well as in the JURKAT cell line, despite its low expression of pY-STAT5, which is an established model for TCR signaling studies. To draw better conclusions on STAT5-correlative activation signals of TCR cascade members, more cell lines and more kinases need to be included in such experiments. We observed a weaker upregulation of TCR pathway genes by hyperactive STAT5A in the absence of RAG2 expression, with delayed T cell neoplasia development compared with more aggressive STAT5B^N642H^-driven disease. Protein crystallization studies have implied that STAT5B^N642H^ mediates structural changes that lead to fortified dimerization accompanied by delayed dephosphorylation adjacent to the SH2-pY interaction and thereby sustains STAT5 activity. Moreover, STAT5B^N642H^ can render cells both cytokine independent or hypersensitive to upstream cytokine stimulation (18). This is consistent with recent findings of the Cools laboratory, showing that cells expressing STAT5B^N642H^ are sensitive to JAK inhibitors, indicating dependency on upstream kinase nodes (65). This concept challenges the classic understanding that pathways signal in one direction. Whether T-ALLs with STAT5 GOF mutations are still cytokine or JAK dependent and if similar crosstalk operates between STAT5 and other kinases, such as ZAP70, ITK, or FYN, remains to be explored.

Previous studies have revealed that T-ALL cells are sensitive to dasatinib, targeting the kinase activity of the TCR pathway member LCK (26, 66). It has been demonstrated through shRNA knockdown of 6 TCR pathway members as well as dasatinib treatment that T-ALL cell lines and patient-derived xenografts are LCK dependent. Moreover, dasatinib synergized with dexamethasone treatment, suggesting combination therapy, including pharmaceutical targeting of T cell kinases, as a promising treatment option (67). It had also been previously shown that 3 T-ALL cell lines were sensitive to ZAP70 knockdown, but the molecular mechanisms of ZAP70 upregulation remained unknown and targeting of ZAP70 has not been tested in T-ALL (67). We confirmed the dependence of T-ALL cells on STAT5B and ZAP70 in a CRISPR/Cas9 genetic knockout experiment and found that STAT5 or SYK inhibitors effectively blocked growth and survival of T-ALL cell lines, more than CD3^+^ T cells from healthy donors. Importantly, the combination of STAT3/5 and ZAP70 inhibitors acted synergistically in T-ALL cell line growth/survival inhibition in vitro and in vivo. JPX-0750 specifically killed T-ALL cells in the vast majority of primary human T-ALL samples and reduced leukemic burden in a T-ALL xenograft alone and more effectively in combination with fostamatinib. The designated STAT3/5 inhibitor degraded STAT5 and STAT3 to similar extents. However, the contribution of STAT3 to T-ALL leukemogenesis is debatable, because no recurrent STAT3 mutations have been found in patients with T-ALL (19), and it is more associated with mature T cell cancers, such as T cell large granular lymphocytic leukemia or NK cell or NK/T cell leukemia (68, 69). Nevertheless, the most likely molecule compensating for STAT5 is STAT3, and thus, dual STAT3/5 degradation could be more effective to fight blood cancers. It remains unclear why fostamatinib affected only 2 of 15 samples. However, higher numbers of samples for testing might be required due to high heterogeneity between T-ALL subtypes, and other kinases might also be involved. Besides SYK/ZAP70, fostamatinib also targets FLT3, LCK, and LYN (according to the manufacturer; Fostamatinib (R788), Syk/FLT3 Inhibitor, MedChemExpress), which might explain the residual sensitivity of T-ALL cells to the inhibitor. In summary, our data reveal how *STAT5A* and *STAT5B* GOF mutations affect thymic T cell development and immature T cell transformation, leading to T-ALL. We uncover potentially novel mechanisms showing that hyperactive STAT5 can directly or indirectly mimic TCR pathway action, and we pioneer the principle of STAT5 or SYK kinase family targeting. We conclude that kinase or direct STAT5 inhibition are promising strategies to block T-ALL growth, but further medicinal chemistry programs and clinical work is needed to move targeted drugs to patients.

## Methods

### Sex as a biological variable.

Both sexes were examined in our mouse experiments, with the exception of the T-ALL xenograft experiment, for which only male mice were used owing to limited availability of female animals. However, previous reports of mice treated with the same compounds indicate equal response of both sexes (55, 68).

### Mice.

All mice used were on a C57BL/6, C57BL/6-Ly5.1 (B6.SJL-*Ptprc^a^Pepc^b^*/BoyCrl), or NSG (NOD.Cg-*Prkdc^scid^ Il2rg^tm1Wjl^*/SzJ) background and maintained in specific pathogen–free conditions in the experimental mouse facility at the University of Veterinary Medicine Vienna. Transgenic mice hemizygous for human *STAT5B^N642H^*, human WT *STAT5B*, or murine *Stat5a*^S710F^ driven by the *Vav1* promoter (70) and 3′ *Vav1* enhancer were generated and genotyped as previously described (16, 17). Double mutants were produced by cross-breeding the 3 transgenic lines with *Rag2^tm1Fwa^* mice and subsequently breeding for homozygosity of the *Rag2*-knockout allele. Due to rapid disease development STAT5B^N642H^ mice were cryopreserved and generated via in vitro fertilization with archived sperm cells as required.

### Flow cytometry.

For flow cytometry, single-cell suspensions were prepared by crushing organs through a 70 μm cell strainer (BD Biosciences). Erythrocytes of spleens were lysed using ammonium-chloride-potassium buffer (150 mM NH_4_CO_3_, 10 mM KHCO_3_, 1 mM EDTA, pH 7.2). All antibodies used for flow cytometry are listed in Supplemental Table 3. All analyses were performed on the BD FACSCanto II using FACSDiva (BD) and FlowJo (version 10.5.3) software. Sorting was performed on a BD FACSAria II.

### Blood analysis.

Mice were anesthetized, and blood was drawn via heart puncture and transferred into EDTA tubes. WBC counts were determined using the scil Vet ABC device.

### RNA-Seq sample acquisition.

DN, DP, and SP8 T cells were harvested from thymi by generating single-cell suspensions and FACS sorting for viable Thy1.2-positive cells with CD4/CD8 expression patterns; this was performed using fluorescent antibodies listed in the Supplemental Table 3. RNA was isolated from snap-frozen cell pellets using the DNA/RNA Allprep Kit (QIAGEN). Details regarding the RNA-Seq processing and analysis protocol are provided in the Supplemental Methods.

### Cell culture.

KOPT-K1 cells were provided by Koshi Akahane from the University of Yamanashi (Chuo, Yamanashi, Japan). All other T-ALL and cell lines were provided in-house. AML and A549 control cell lines were purchased from DMSZ. All cells were cultivated with complete RPMI 1640 or DMEM medium (10% FCS, 2 mM L-glutamine, 10 U/mL penicillin-streptomycin) (all from Gibco, Thermo Fisher Scientific). The cell lines were authenticated and regularly tested for *Mycoplasma* using the MycoAlert PLUS detection kit by Lonza (LT07-710).

### Western blot.

Immunoblotting was performed using standard protocols and antibodies as listed in Supplemental Table 4. Images of membranes were obtained using IRDye fluorescent secondary antibodies and an Odyssey CLx imaging system (LI-COR). Signal quantification was performed using Image Studio Lite (version 5.2).

### Immunohistochemistry.

Mouse organs were processed and analyzed using standard protocols; details and antibody specifications are provided in the Supplemental Methods.

### Transplantation experiments.

STAT5B^N642H^ mice were sacrificed, and 10^6^ viable CD3^+^CD8^+^ lymphocytes (from LNs) or Thy1.2^+^ CD4^+^ CD8^+^ DP thymocytes (from thymus) were isolated by flow cytometry and transplanted into Ly5.1 or C57BL/6Nrj recipients via tail vein injection. Recipients were monitored for signs of disease daily and sacrificed at indicated time points or at a moribund state.

### Human database analyses.

Human gene expression data were extracted and downloaded from the Haferlach Leukemia, Zhang Leukemia, or Andersson Leukemia data sets using the Oncomine Research Premium Edition database (71). Human cell line gene expression data were downloaded from DepMap (https://depmap.org/portal/home/#/).

### Patient analysis.

DNA and RNA were isolated from total leukocytes of primary T-ALL patient samples, and whole-genome sequencing and RNA-Seq was performed as described previously (72).

### Sanger sequencing.

Cells were harvested, RNA was isolated using the AllPrep DNA/RNA Mini Kit from QIAGEN, and cDNA was generated using the RevertAid First Strand cDNA Synthesis Kit from Thermo Fisher Scientific. A sequence spanning over the mRNA region encoding the STAT5B C-terminal region was amplified by PCR (forward primer, GGCAATGGTTTGACGGTG; reverse primer, GGATCCACTGACTGTCCATT). The 646 bp PCR product was separated on an agarose gel using standard conditions. DNA was isolated using the MinElute PCR Purification Kit (QIAGEN), and Sanger sequencing was performed by Microsynth Austria GmbH (primer GCCTCATTGGAATGATGG).

### ChIP-Seq and initial processing.

ChIP-Seq was performed as previously described (73). The antibodies used for each experiment are listed in the Supplemental Table 5. For each ChIP, 5 μg antibody coupled to 2 μg magnetic Dynabeads (Life Technologies) was added to 3 mL sonicated nuclear extract from formaldehyde-fixed cells. Chromatin was immunoprecipitated overnight, cross-links were reversed, and DNA was purified by precipitation with phenol/chloroform/isoamyl alcohol. DNA pellets were resuspended in 25 μL TE buffer. Illumina sequencing, library construction, and ChIP-Seq analysis methods were previously described (73). The ChIP-Seq analysis is further described in the Supplemental Methods.

### CRISPR/Cas9 knockout.

CRISPR/Cas9-mediated knockout of *STAT5B* and *ZAP70* in KOPT-K1, DND-41, JURKAT, and LOUCY cell lines was performed as previously described (74). Briefly, cells were lentivirally transduced with a Cas9 construct (lentiCas9-Blast, https://www.addgene.org/52962/) and selected with Blasticidin (Eubio, ant-bl-1, 10 μg/mL final concentration). Upon stable CAS9 expression, cells were lentivirally transduced with a LentiGuide-Puro-IRES-GFP backbone containing respective guide RNAs coupled to GFP expression (Supplemental Table 6), resulting in a mixed population of GFP-positive and -negative cells. The percentage of GFP-positive cells was monitored over time on the IQue3 (BioScience, Sartorius Group) and normalized to day 3 after infection and to the nontargeting control *AAVS1*. The depletion of cells over time with the knockout of the positive control *RPL17* verifies a functional CAS9 protein.

### In vitro drug treatments.

CD3^+^ T cells from healthy donors were isolated from blood, using the MojoSort Human CD3 T cell isolation kit (BioLegend). 10^4^ (cell lines) or 10^5^ (human CD3 controls) cells per well were seeded in triplicates in a flat-bottom 96-well plate. On the subsequent day, cells were treated with serial 1:2 dilutions of the drug of interest. Bortezomib (MedChemExpress, HY-10227), a proteasome blocker, served as positive control and DMSO served as negative control. DMSO concentrations were equal in all wells to exclude effects by variations in concentrations of the vehicle. Treated cells were incubated at 37°C and 5% CO_2_ for 72 hours. Cell viability was assessed by using the CellTiter-Glo Luminescent cell Viability Assay (Promega) or the CellTiter-Blue Cell Viability Assay (Promega) and the GloMax Discover Microplate Reader (Promega). IC_50_ values were calculated from ATP luminescence or resorufin fluorescence using nonlinear regression of log-transformed, normalized data.

For Western blot analysis, 1.5 × 10^6^ cells were seeded in 2 mL medium per well in 6-well plates. On the next day, cells were treated with indicated doses of JPX-0750, fostamatinib (MedChemExpress, HY-12067), or gusacitinib (MedChemExpress, HY-103018). After 24 hours, cells were harvested into 15 mL tubes, washed with ice-cold PBS, and processed for Western blot analysis.

### Drug synergy testing.

The drug combination synergy analysis was performed using the SynergyFinder 2.0 web application. The degree of synergy was quantified using the zero interaction potency (ZIP) model, which captures the drug interaction relationships by comparing the change in the potency of the dose-response curves between individual drugs and their combinations. In addition to the overall ZIP score over the dose-response matrix, the SynergyFinder web tool (75) enabled calculation of the most synergistic area (MSA) score, which represents the most synergistic 3-by-3 dose-window. According to the ZIP model, an MSA score below –10 indicates that the 2 drugs have antagonistic effects, a score between –10 and 10 indicates an additive effect, and a MSA score above 10 indicates a synergistic effect. The 4-parameter logistic regression model was used as the curve-fitting algorithm in the SynergyFinder web tool. All assays were performed in 2 biological replicates.

### Chemosensitivity profiling in primary patient cells.

Bone marrow aspiration and peripheral blood samples from patients with T-ALL were obtained from the Division of Hematology and Hemostaseology at the Medical University of Vienna. Each sample was frozen after Ficoll-Paque mononuclear cell separation. Fostamatinib and JPX-0750 were printed on tissue culture-treated 384-well plates (Corning) using an Echo 550 (Labcyte Inc.) acoustic dispenser with 5 different concentrations in 10-fold dilutions encompassing a 10,000-fold concentration range (1–10,000 nM) in duplicates (only 100, 1,000, and 10,000 nM responses were taken into analysis). DMSO was used as a negative control. Samples were thawed, resuspended in RPMI + 10% FBS, and seeded at a concentration of 4 × 10^5^ to 5 × 10^5^ per mL (50 mL/well) onto predrugged 384-well plates. The cells with drugs were incubated at 37°C for 24 hours. After incubation, the cells were spun down (100*g* for 5 min), and the supernatant from each well was aspirated using the Biotek MultiFlo FX Multi-Mode Dispenser. The cells were stained with DAPI and antibodies marking T-ALL or control cells (Supplemental Table 3) at a 1:500 dilution in PBS for 1 hour at room temperature. Then, the plates were run on an iQue3 high-throughput flow cytometer screener (Sartorius), which facilitated multiplex cell analysis and subclone-specific drug screening. The data were gated to remove noise, doublets, and dead cells. T-ALL cells from each sample were determined by evaluating histological reports and FACS marker positivity.

### Xenograft experiment.

10^6^ KOPT-K1 or DND-41 cells were transplanted via tail vein injection into male 12- to 16-week-old NSG (NOD.Cg-*Prkdc^scid^ Il2rg^tm1Wjl^*/SzJ) recipients. Transplanted cells were traced by regular bleedings and subsequent flow cytometry staining for surface human CD45 to verify engraftment in all mice used for the treatment. Recipients were randomized and treated daily with intraperitoneal injection of JPX-0750 at a dosage of 10 mg/kg, a combination of JPX-0750 at 10 mg/kg and fostamatinib at 80 mg/kg, or vehicle for 21 subsequent days, starting 12 days (KOPT-K1) or 20 days (DND-41) after injection. In the combination cohort, 2 mice died during treatment due to toxic side effects. Both inhibitors were dissolved in DMSO and diluted 1:10 in 50% PEG-300, 5% TWEEN 80, 40% PBS. In the combination group of the KOPT-K1 xenograft, one blood sample was clotted and therefore excluded from analysis.

### Statistics.

Statistical tests used are indicated in the figure legends and include unpaired 2-tailed Student’s *t* test, Mann-Whitney *U* test, 1-way ANOVA with Dunnett’s multiple comparison test, and 1-way ANOVA with Tukey’s multiple comparison test. *P* values of less than 0.05 were considered significant.

### Study approval.

All mouse experiments were approved by the institutional ethics committee of the University of Veterinary Medicine Vienna and licensed under BMWF-68.205/0023-II/3b/2014, BMWFW-68.205/0166-WF/V/3b/2015, BMBWF-68.205/0130-WF/V/3b/2016, and BMBWF-68.205/0041-V/3b/2019 by the Austrian Federal Ministry of Education, Science, and Research.

Material from patients was obtained with approval from the ethics committees of the Medical University of Vienna (EK 1727/2022). Research was conducted in accordance with the Declaration of Helsinki.

Tissue samples used for immunohistochemistry were provided by the University Cancer Center Frankfurt, Frankfurt, Germany. Written informed consent was obtained from all patients in accordance with the Declaration of Helsinki, and the study was approved by the institutional review boards of the University Cancer Center Frankfurt and the ethics committee at the University Hospital Frankfurt, Frankfurt, Germany (project SHN-06-2018).

### Data availability.

All data that were used for analyses in this study can be requested from the corresponding author. RNA-Seq and ChIP-Seq data were uploaded to the GEO database repository (GSE218858 and GSE219155). Values for all data points in graphs are reported in the Supporting Data Values file.

## Author contributions

RM designed and supervised the study. T Suske, HS, GM, NP, MWZ, DIA, BM, SS, KS, AP, TP, CS, DP, EH, DJ, TAM, MMKA, AO, HTTP, and KZ designed, performed, and/or analyzed experiments. FR, MWZ, and TE contributed bioinformatics support. MD, AB, and TR generated and maintained transgenic mice. TK, CB, MM, RF, EDDA, PTG, TA, SM, T Sanda, SH, FG, GH, TH, PBS, HAN, ATL, and MH contributed to the interpretation of data and revised the manuscript. RM and T Suske wrote the manuscript.

## Supplementary Material

Supplemental data

Unedited blot and gel images

Supporting data values

## Version 1 04/15/2024

Electronic publication
