# Supplementary material for: Hyperactive STAT5 hijacks T cell receptor signaling and drives immature T cell acute lymphoblastic leukemia
Source: J Clin Invest. 2024 Apr 15;134(8):e168536. doi: 10.1172/JCI168536 (PMC11014662; doi:10.1172/JCI168536)
Supplement: Supplemental data [file jci-134-168536-s148.pdf]

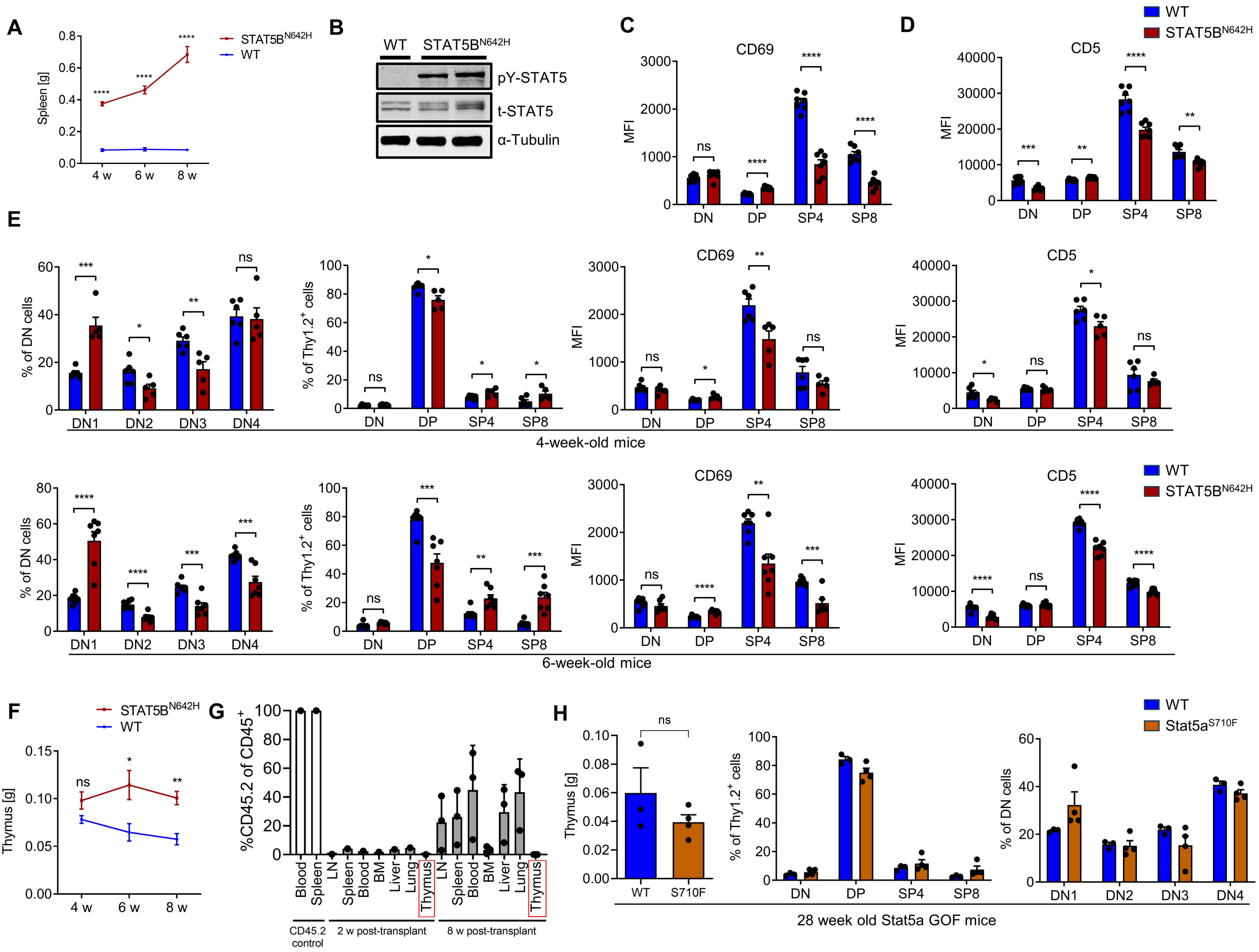

Supplemental Figure S1. STAT5B^{N642H} causes more drastic alterations in thymic development than Stat5a^{S710F}. A, Spleen weights in g from four-, six- and eight-week-old STAT5B^{N642H} or WT littermate mice. **B**, Western blot analysis for pY-STAT5, t-STAT5 expression and Tubulin as loading control in thymi from WT and STAT5^{N642H} mice. **C** and **D**, Mean fluorescent intensity (MFI) of CD69 and CD5 in DN, DP, SP4 and SP8 cells **E**, DN1, DN2, DN3, DN4 (CD25/CD44) and DN, DP, SP4, SP8 (CD4/CD8) cells gated on Thy1.2^{+} thymocytes of four- (upper panel) and six- (lower panel) week-old mice and MFI of CD69 and CD5 in DN, DP, SP4, SP8 stages. **F**, Thymus weights in g from four-, six- and eight-week-old STAT5B^{N642H} or WT littermate mice, n for all groups = 5 or more, each experiment was performed twice independently. **G**, Percentages of CD45.2^{+} cells in hematopoietic organs of CD45.1 recipient mice, 2 (n = 1) or 8 weeks (n = 3) after intravenous transplantation of sorted CD8^{+} cells from LNs of terminally diseased STAT5B^{N642H} transgenic mice on CD45.2 background. Blood and spleen cells from a CD45.2 mouse were used as positive control. **H**, Thymus weights of 13- and 28-week-old Stat5a^{S710F} mice or WT littermates and flow cytometry analysis as described above from these mice. **A**, **C**, **D**, **E** and **F**, Significant differences are indicated as $^{*}P < 0.05$, $^{**}P < 0.01$, $^{***}P < 0.001$, $^{****}P < 0.0001$, by two-way Anova (**A** and **F**) or unpaired two-tailed Student's t-test (**C**, **D** and **E**). Error bars show mean +/-SEM.

Suppl. Fig. 1

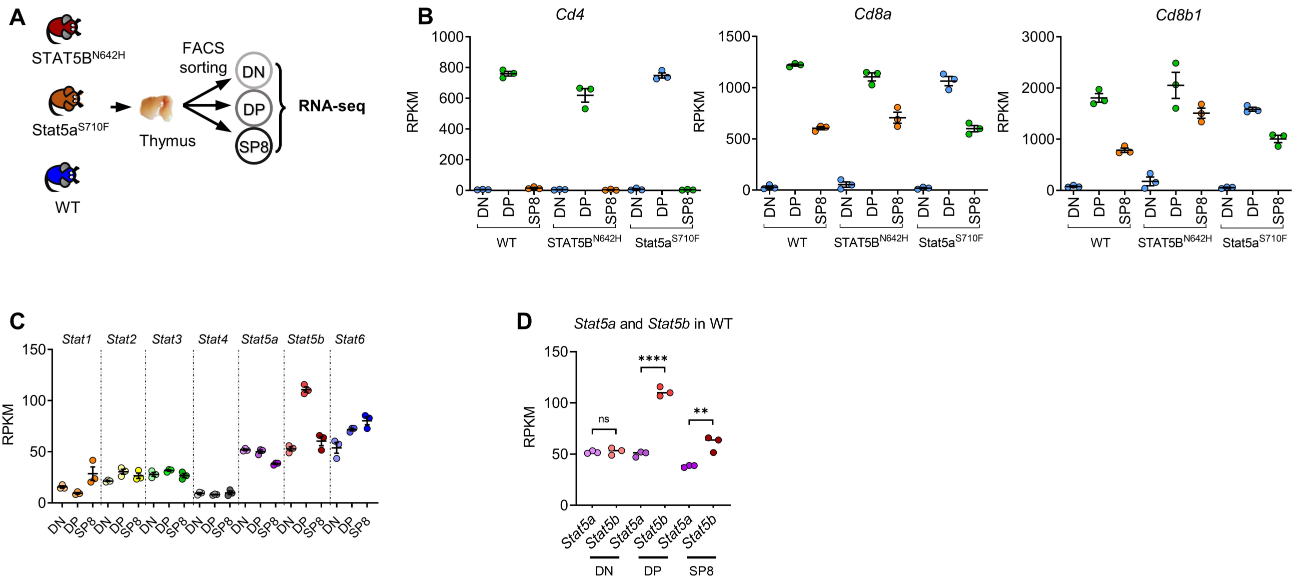

Supplemental Figure S2. Validation of RNA-seq sample purity and GO-term analysis. A, Schematic depiction of thymocyte sample acquisition and RNA-seq in biological triplicates. Single cell suspensions of thymi were sorted for DN, DP and SP8 T-cell progenitor subsets by flow cytometry and subsequently subjected to RNA-seq. **B**, RPKM values of *Cd4*, *Cd8a* and *Cd8b1* confirming purity of sorted DN, DP and SP8 cells from WT, STAT5B^{N642H} and Stat5a^{S710F} thymi. **C**, RPKM values of all STAT family members in WT DN, DP and SP8 thymocytes. **D**, *Stat5a* and *Stat5b* mRNA expression in WT thymocytes. In **C**, Significant differences are indicated as $^{**}P < 0.01$, $^{****}P < 0.0001$, by unpaired two-tailed Student's t-test. In **B**-**D**, error bars show mean +/-SEM.

Suppl. Fig. 2

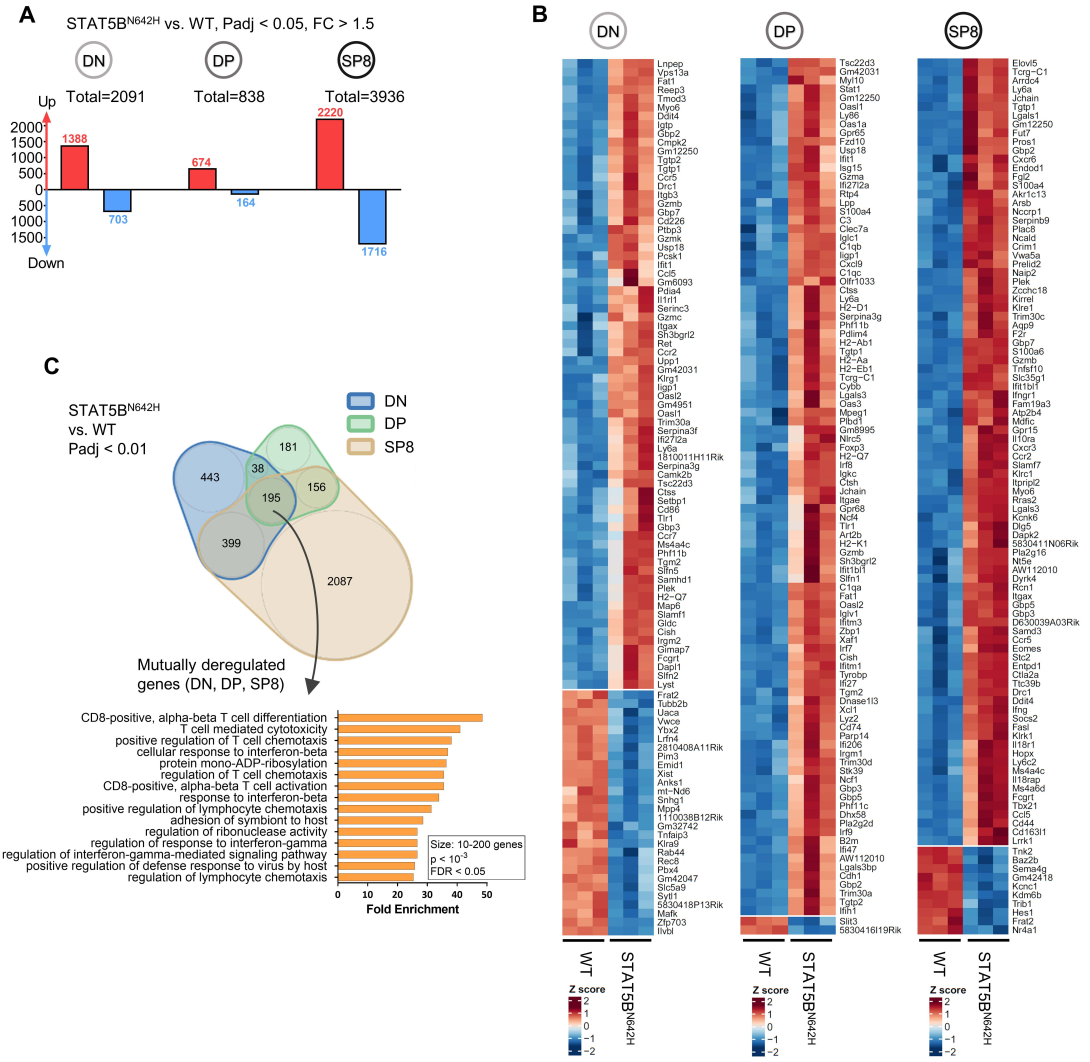

Supplemental Figure S3. STAT5B^{N642H} leads to to high transcriptional activity and an activated T-cell phenotype. A, Number of up- or downregulated genes in DN, DP and SP8 cells of STAT5B^{N642H} vs. WT thymocytes (*Padj*<0.05, fc>1.5). **B**, Heatmap of the top 100 deregulated genes of these comparisons, log10 transformed normalized counts from DESeq2 were used for this analysis. **C**, Top: Venn diagram showing deregulated genes (STAT5B^{N642H} vs. WT) in DN, DP and SP8 T-cells and overlaps between subgroups, only genes with *Padj*<0.01 from respective DESeq comparisons were taken into analysis. Bottom: fold enrichment values from GO-term analysis of 195 mutually deregulated genes between all subsets, GO-terms containing 10-200 genes, $P < 10^{-3}$ and false discovery rate (FDR) <0.05 as inclusion criteria were taken into analysis.

Suppl. Fig. 3

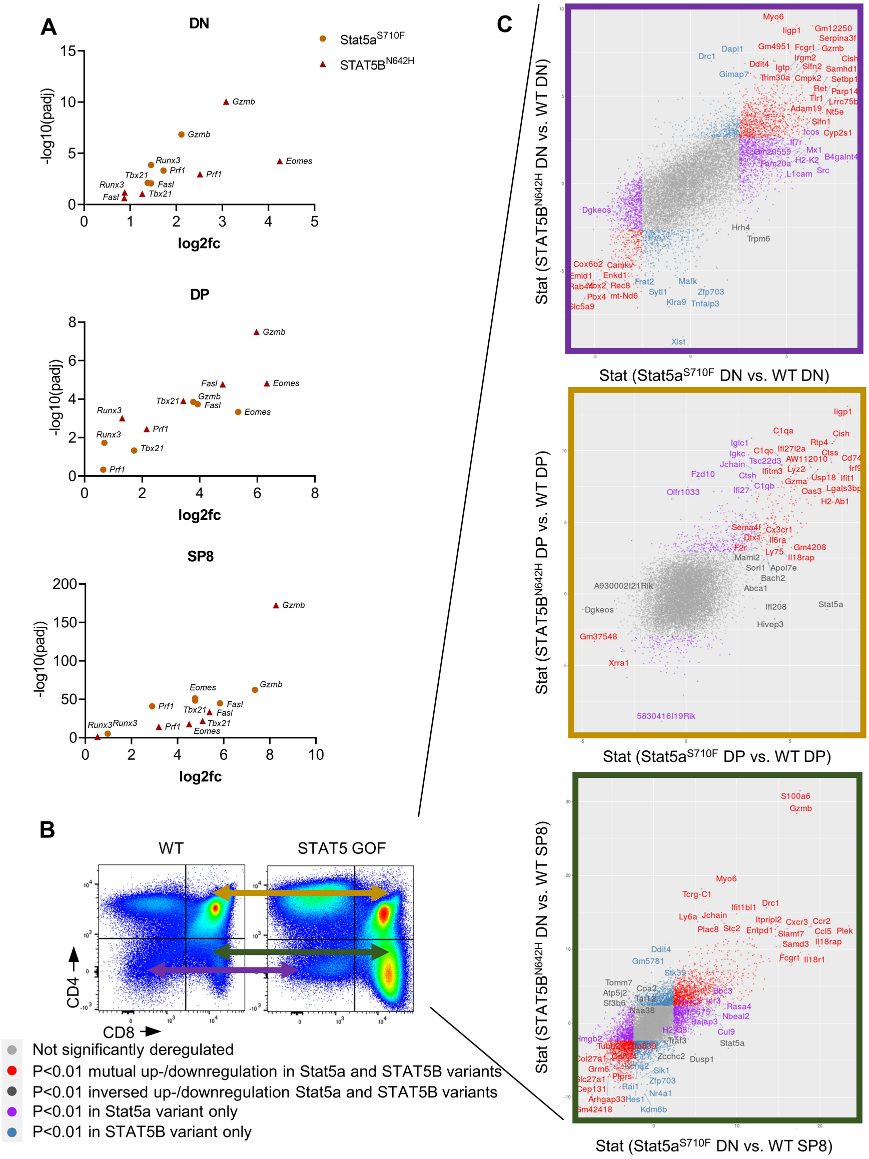

Supplemental Figure S4. STAT5B^{N642H} and Stat5a^{S710F} mice share similar transcriptional deregulation in the thymus. A, *Padj* and log2fc of genes involved in T-cell activation or lineage commitment in STAT5B^{N642H} and Stat5a^{S710F} thymic subsets. Values determined by DESeq analysis. **B**, Representative flow cytometry plots indicating comparisons of thymic subsets by RNA-seq. **C**, Scatterplots showing Stat-values determined by DESeq analysis of deregulated genes of STAT5B^{N642H} vs. WT on the y-axes and Stat5a^{S710F} vs. WT on the x-axes in respective thymic subsets, as indicated in **B**.

Suppl. Fig. 4

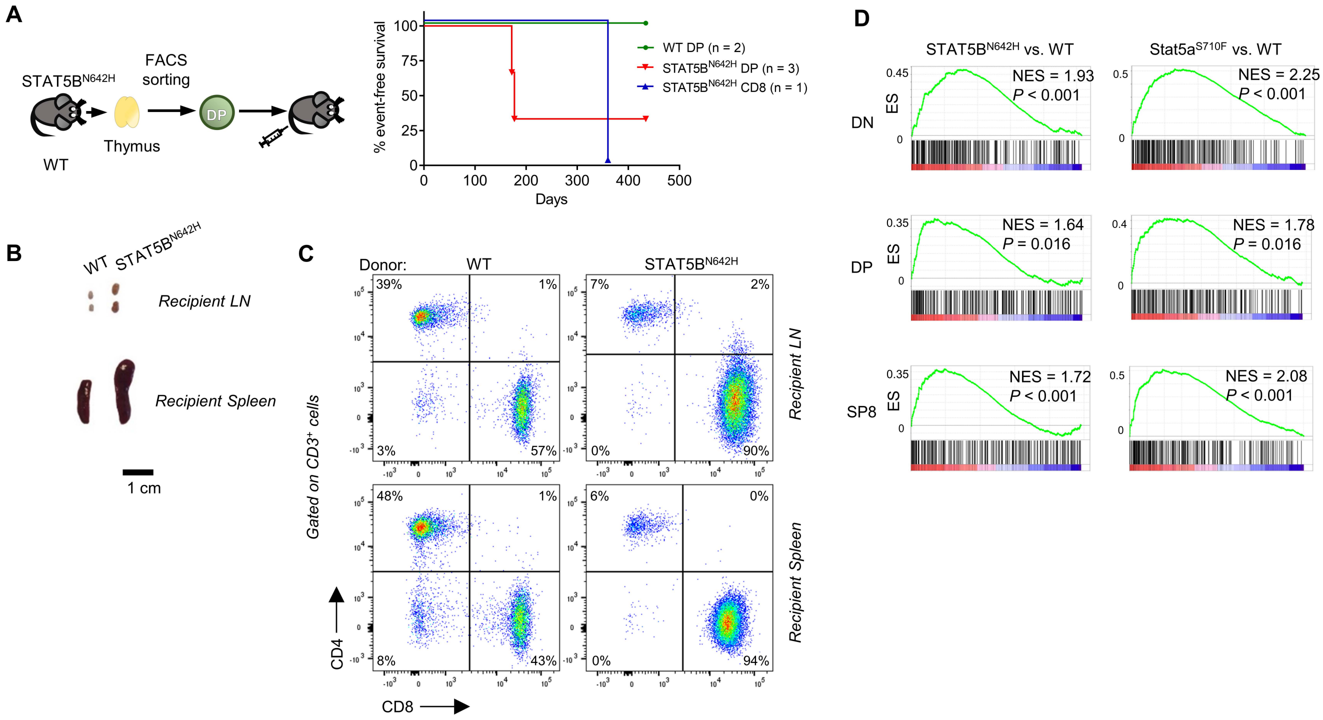

Supplemental Figure S5. Transplanted DP cells from thymi of STAT5B^{N642H} mice give rise to CD8^{+} mature T-cell lymphoma. A, Schematic representation and Kaplan-Meier event-free survival plot of recipient mice transplanted with DP thymocytes from WT or STAT5B^{N642H} donor mice or CD8^{+} T-cells from LNs of STAT5B^{N642H} mice as positive control. **B**, Representative images of spleens and LNs of DP thymocyte recipients at terminal stage. **C**, CD4 and CD8 surface expression by flow cytometry analysis, gated on CD3^{+} cells of LNs and spleens from recipients of DP thymocytes. **D**, GSEA comparing top 250 upregulated genes in human ETP-ALL (reference 10) to deregulated genes in STAT5B^{N642H} DN, DP or SP8 vs. respective WT populations, NES: normalized enrichment score.

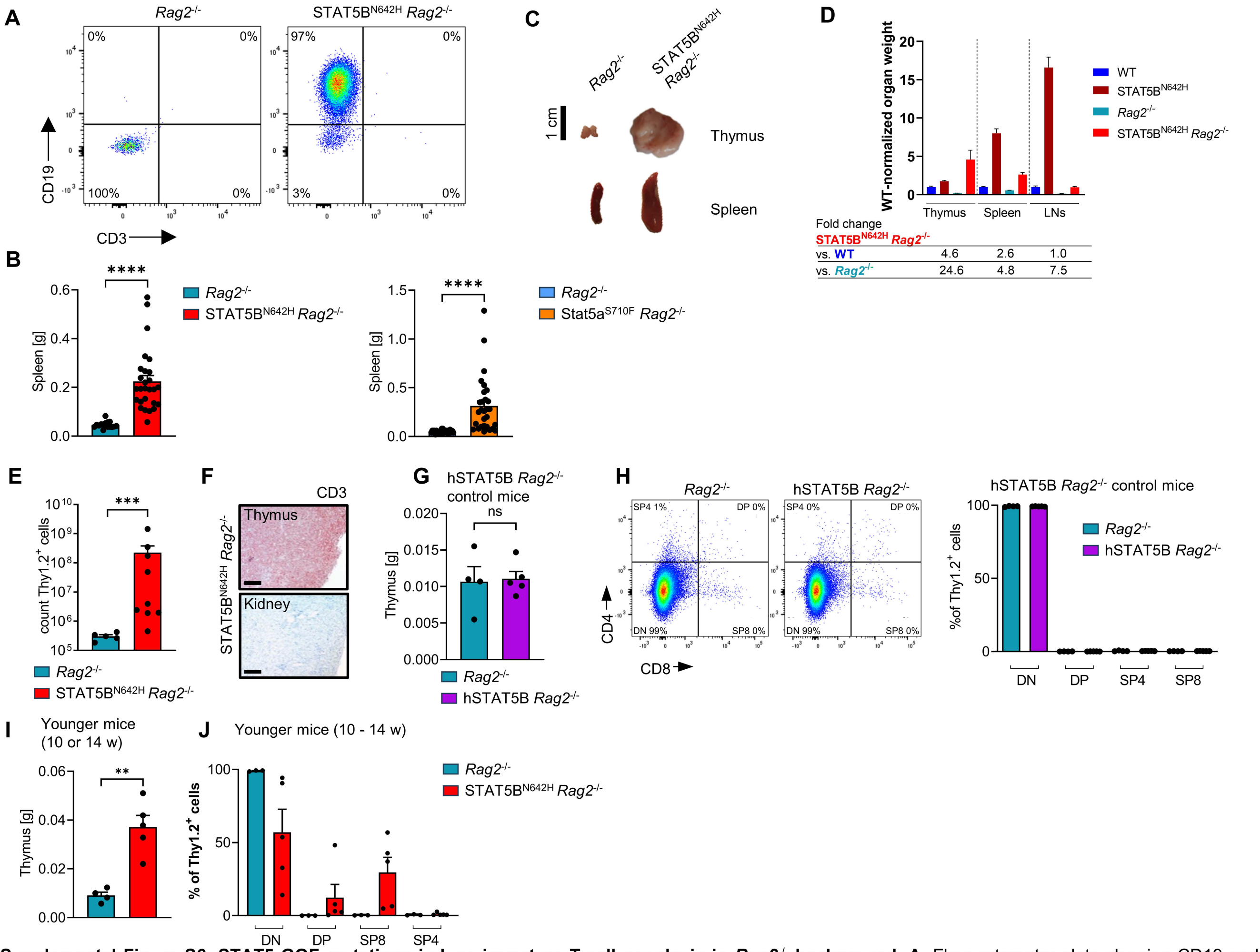

Supplemental Figure S6. STAT5 GOF mutations induce immature T-cell neoplasia in *Rag2*$^{-/-}$ background. A, Flow cytometry plots showing CD19 and CD3 surface expression in the LN of one STAT5B^{N642H} *Rag2*$^{-/-}$ mouse that developed a B-cell lymphoma and a *Rag2*$^{-/-}$ mouse as control. **B**, Spleen weights in g of diseased STAT5B^{N642H} *Rag2*$^{-/-}$ (n = 27) or Stat5a^{S710F} *Rag2*$^{-/-}$ (n = 29) vs. respective *Rag2*$^{-/-}$ littermates (n = 17/24). **C**, Representative images of thymi and spleens of *Rag2*$^{-/-}$ and diseased STAT5B^{N642H} *Rag2*$^{-/-}$ mice. **D**, Relative organ weights (thymus, spleen, LN) of WT, STAT5B^{N642H}, *Rag2*$^{-/-}$ and STAT5B^{N642H} *Rag2*$^{-/-}$ normalized to WT. Fold changes of STAT5B^{N642H} *Rag2*$^{-/-}$ compared to WT or *Rag2*$^{-/-}$ are shown below, n ≥ 7 for all organs/genotypes **E**, Absolute counts for Thy1.2^{+} cells in thymi of STAT5B^{N642H} *Rag2*$^{-/-}$ (n = 9) and *Rag2*$^{-/-}$ littermates (n = 5). **F**, Representative immunohistochemical CD3 staining in the thymus of STAT5B^{N642H} *Rag2*$^{-/-}$ mice and kidney as negative control tissue, original magnification: 10x, scale bar = 200 µm. **G**, Thymus weights in g of WT human (h)STAT5B *Rag2*$^{-/-}$ mice and *Rag2*$^{-/-}$ littermates. **H**, Representative flow cytometry plots and quantification of CD4/CD8 staining gated on Thy1.2^{+} cells in *Rag2*$^{-/-}$ and WT human (h) STAT5B *Rag2*$^{-/-}$ mice. **I**, Thymus weights in g of non-terminal-diseased STAT5B^{N642H} *Rag2*$^{-/-}$ mice and *Rag2*$^{-/-}$ littermates of 10-14 weeks of age. **J**, Analysis of CD4/CD8 staining gated on Thy1.2^{+} cells in non-terminal-diseased STAT5^{N642H} *Rag2*$^{-/-}$ mice and *Rag2*$^{-/-}$ littermates of 10-14 weeks of age. In **B**, **E** and **I**, significant differences are indicated as $^{**}P < 0.01$, $^{***}P < 0.001$, $^{****}P < 0.0001$, by a Mann-Whitney U test. All error bars show mean +/-SEM.

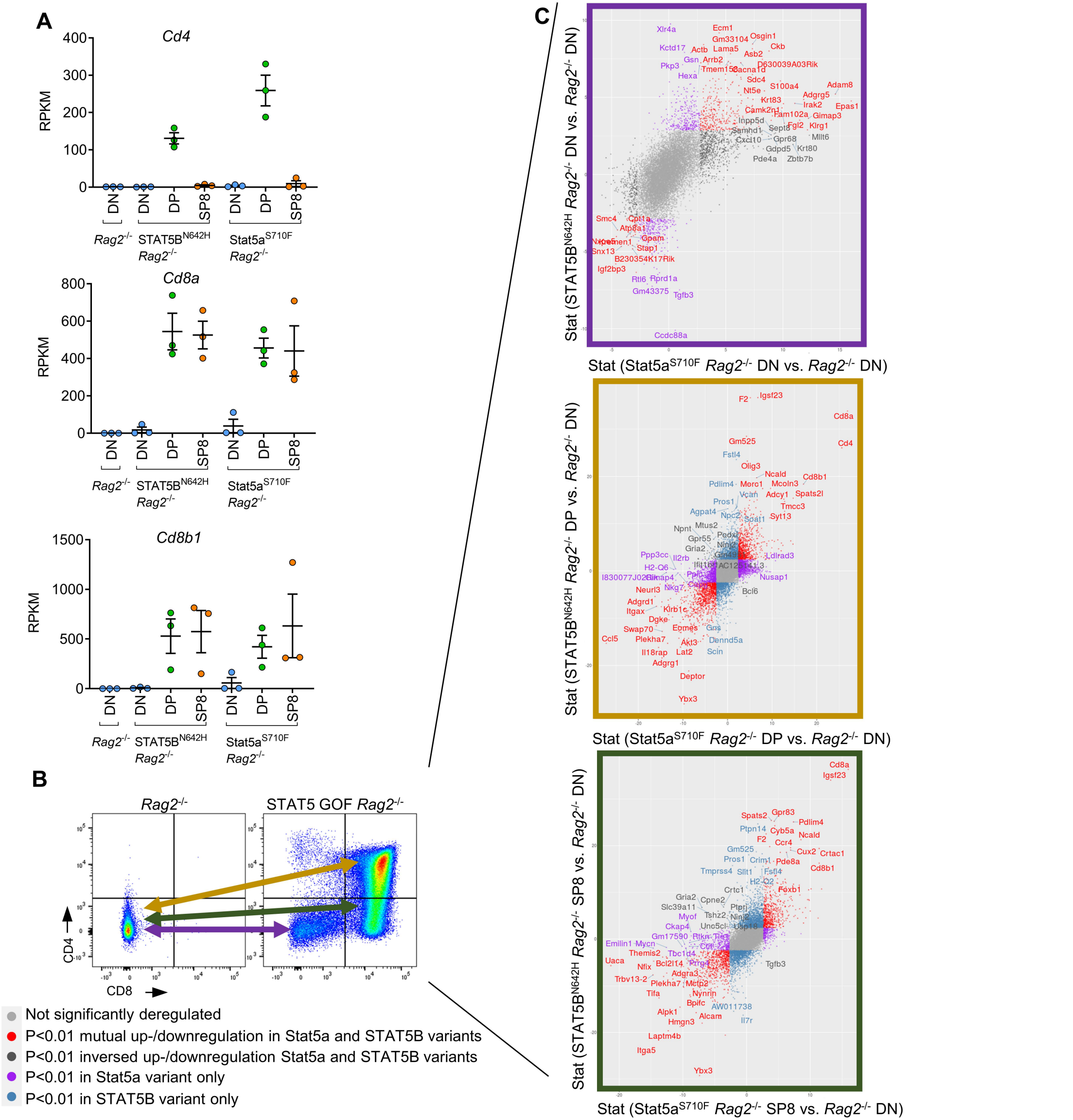

Supplemental Figure S7. Thymic neoplasms of STAT5B^{N642H} *Rag2$^{-/-}$* and Stat5a^{S710F} *Rag2$^{-/-}$* mice display similar transcriptomic changes. A, RPKM values of *Cd4*, *Cd8a* and *Cd8b1* confirming purity of sorted DN, DP and SP8 cells from *Rag2$^{-/-}$*, STAT5B^{N642H} *Rag2$^{-/-}$* and Stat5a^{S710F} *Rag2$^{-/-}$* thymi. **B**, Representative flow cytometry plots indicating comparisons of thymic subsets by RNA-seq. **C**, Scatterplots showing Stat-values determined by DESeq of deregulated genes of STAT5B^{N642H} *Rag2$^{-/-}$* vs. *Rag2$^{-/-}$* on the y-axes and Stat5a^{S710F} *Rag2$^{-/-}$* vs. *Rag2$^{-/-}$* on the x-axes in respective thymic subsets, as indicated in **B**. In **A**, error bars show mean +/-SEM.

Suppl. Fig. 7

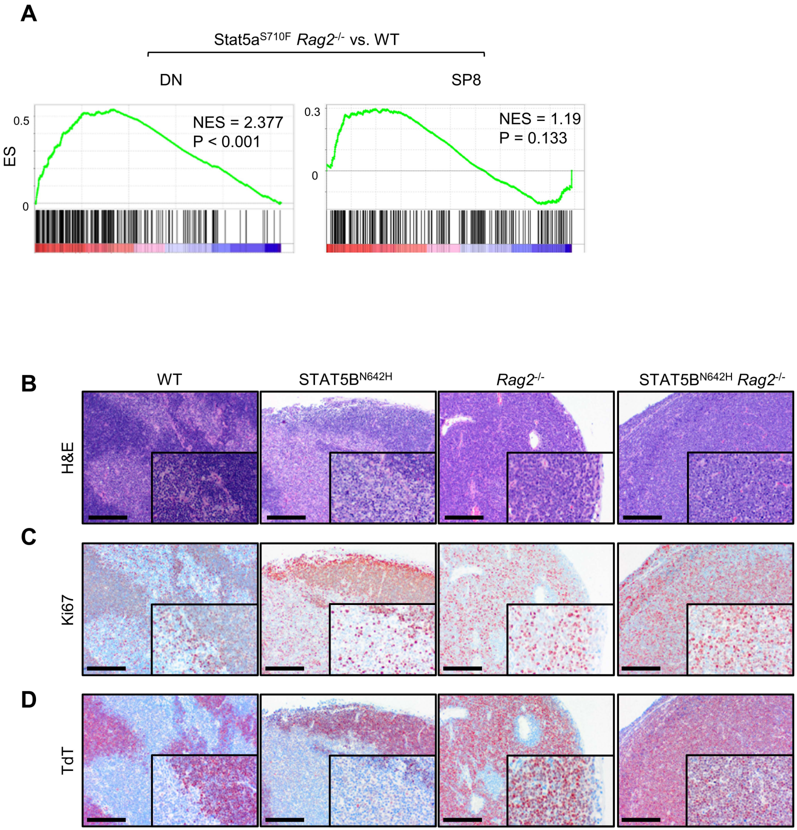

Supplemental Figure S8. STAT5B^{N642H} *Rag2$^{-/-}$* thymic neoplasm phenotypically resemble human T-ALL. A, GSEA comparing top 250 upregulated genes in human ETP-ALL (reference 10) to deregulated genes in Stat5a^{S710F} *Rag2$^{-/-}$* DN or SP8 vs. respective WT populations, NES: normalized enrichment score. Due to low transcriptional association with ETP-ALL genes, DP cells were not displayed in the analysis. **B**-**D**, Histological analysis of thymi from WT, STAT5B^{N642H}, *Rag2$^{-/-}$* and STAT5B^{N642H} *Rag2$^{-/-}$* mice, staining for H&E (**B**), anti-Ki67 (**C**) and TdT (**D**), representatives of at least 3 biological replicates, original magnifications: 10x and 20x (insets), scale bar = 500 µm.

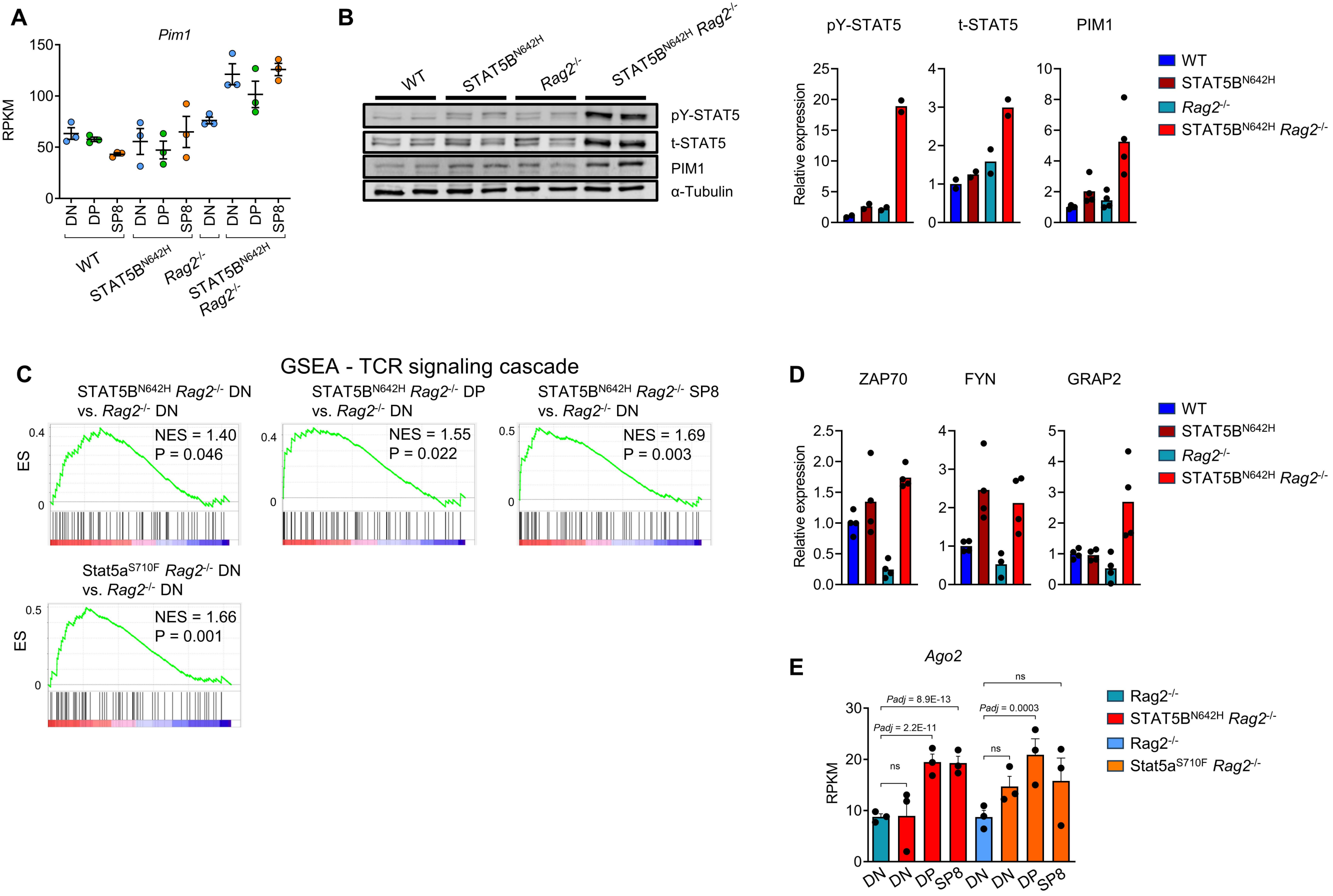

Supplemental Figure S9. STAT5B^{N642H} *Rag2*$^{-/-}$-induced neoplasms express highly activated STAT5 and its downstream oncogene PIM1. A, RPKM values of *Pim1* from RNA-seq in DN, DP and SP8 cells of WT, STAT5B^{N642H}, *Rag2*$^{-/-}$ and STAT5B^{N642H} *Rag2*$^{-/-}$ thymi. **B**, Western blot analysis for pY-STAT5, t-STAT5 and PIM1 expression from whole cell extracts of thymocytes from WT, STAT5B^{N642H}, *Rag2*$^{-/-}$ and STAT5B^{N642H} *Rag2*$^{-/-}$ mice (n = 2 for pY-t-STAT5 and n = 4 for PIM1, each genotype) and quantification thereof. **C**, GSEA comparing indicated contrasts to 70 genes downstream of TCR signaling, NES: normalized enrichment score. **D**, Relative signals to loading controls obtained from Western blot analysis for ZAP70, FYN and GRAP2 expression in thymi of mice of indicated genotypes (n = 4 each genotype). **E**, RPKM values of *Ago2* DN, DP, SP8 (STAT5B^{N642H} *Rag2*$^{-/-}$ and Stat5a^{S710F} *Rag2*$^{-/-}$) cells vs. DN cells (respective *Rag2*$^{-/-}$ littermates), *Padj* determined by DESeq analysis. In **A**, **B**, **D** and **E**, error bars show mean +/-SEM.

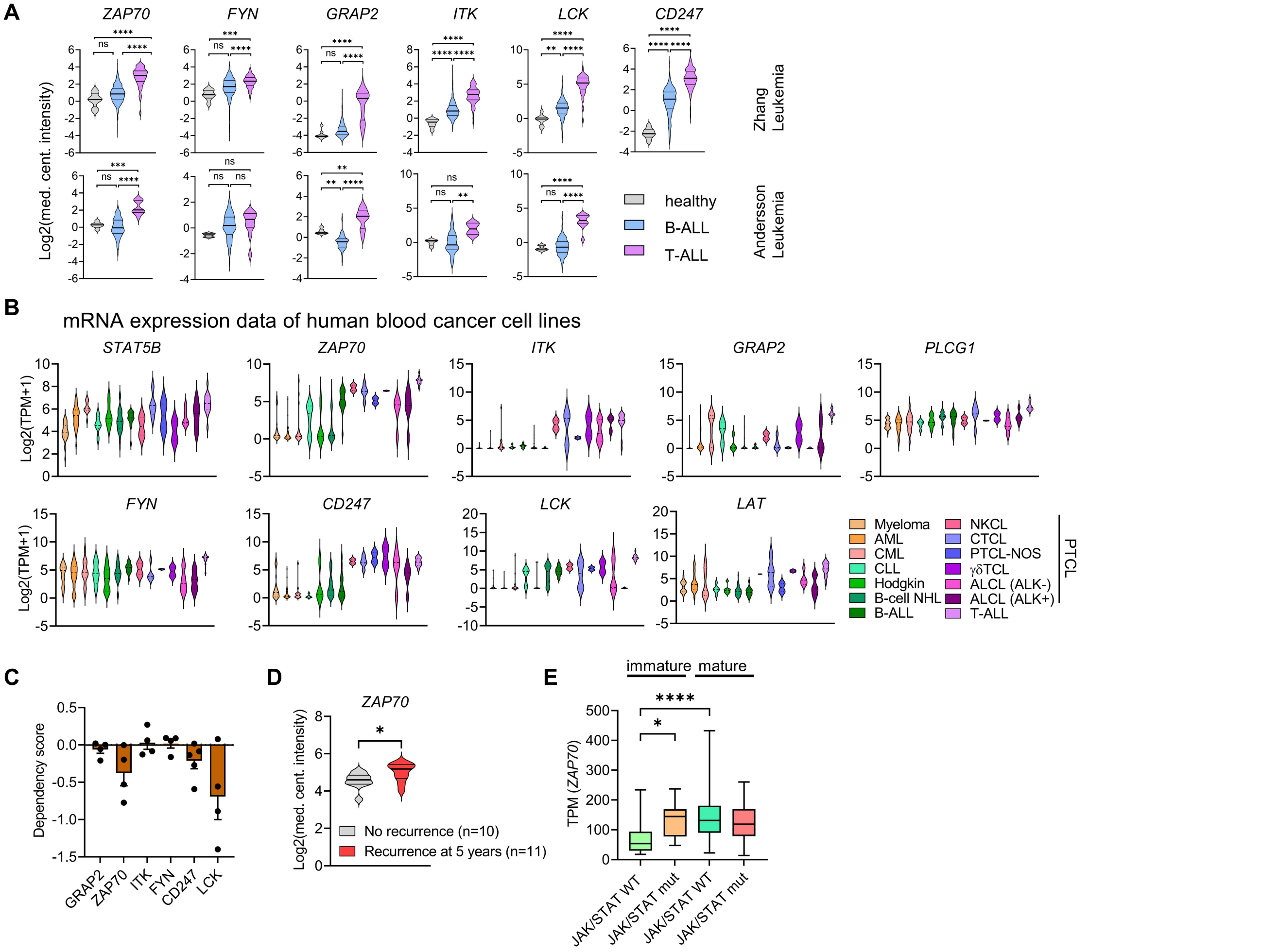

Supplemental Figure S10. TCR pathway genes are highly expressed in human T-ALL. A, mRNA expression data of human patients suffering from B-ALL (n = 483) or T-ALL (n = 84) and healthy bone marrow control (n = 8) cells. Data extracted from the “Andersson Leukemia” and “Zhang Leukemia” studies of the Oncomine database. **B**, Expression of *STAT5B*, *ZAP70*, *ITK*, *GRAP2*, *PLCG1*, *FYN*, *CD247*, *LCK* and *LAT* mRNA in human blood cancer cell lines. Data extracted from DepMap (www.depmap.org). TPM: transcripts per million. Abbreviations and numbers in brackets: Myeloma (31), AML: acute myeloid leukemia (44), CML: chronic myelogenous leukemia (15), CLL: chronic lymphocytic leukemia (6), Hodgkin (8), B-cell NHL: non-Hodgkin lymphoma (51), B-ALL: B-cell acute lymphoblastic leukemia (19), NKCL: natural killer cell lymphoma (2), CTCL: cutaneous T-cell lymphoma (5), PTCL-NOS: peripheral T-cell lymphoma, not otherwise specified (2), ALCL: anaplastic large cell lymphoma (ALK$^-$: 5, ALK$^+$: 6), T-ALL (16). **C**, Dependency scores determined of indicated genes in T-ALL cell lines. Data for **B** and **C** extracted from DepMap (www.depmap.org). **D**, *ZAP70* expression of T-ALL patients with no relapse vs. patients with relapse after 5 years. Data extracted from the Oncomine database. **E**, *ZAP70* expression in TPM in immature or mature T-ALL patients, with or without activating mutations in *IL7R*, *JAK1*, *JAK3*, *STAT5A* or *STAT5B*. In **D**, significant differences are indicated as *$P < 0.05$, by a Mann-Whitney U test. In **A** and **E**, significant differences are indicated as *$P < 0.05$, **$P < 0.01$, ***$P < 0.001$, ****$P < 0.0001$ by one-way Anova with Dunnet’s multiple comparison test. Error bars show mean +/-SEM.

Suppl. Fig. 10

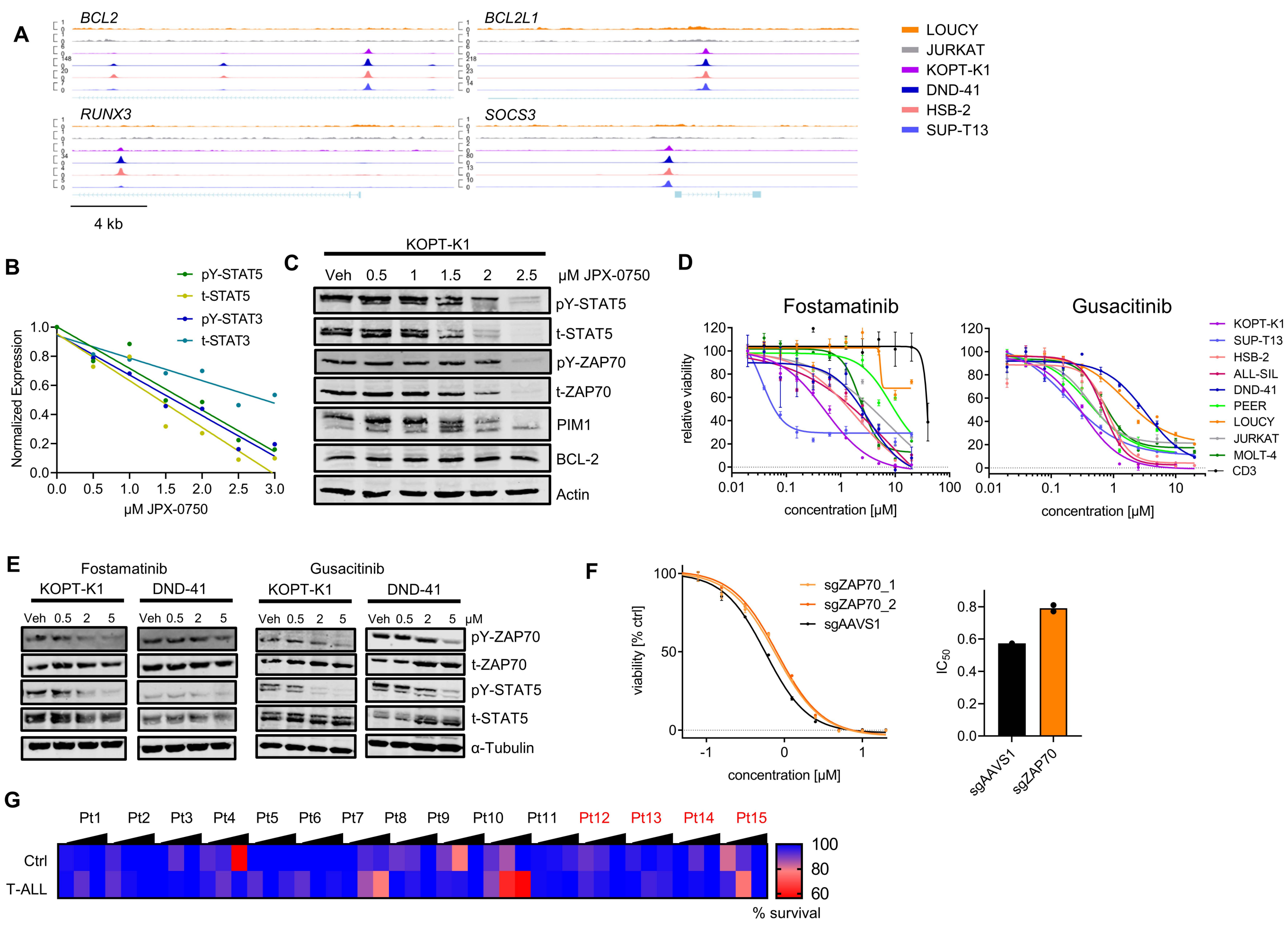

Supplemental Figure S11. STAT5 binds to *bona fide* target genes in cell lines with high STAT5 activation and its target genes represent valuable targets in T-ALL cells. A, ChIP-seq lanes indicating STAT5B binding at the promoter region of genes that were previously confirmed as STAT5 target genes, indicated in six T-ALL cell lines, CPM indicated on the Y-axis. **B**, Quantification of pY-STAT5/-STAT3, t-STAT5/-STAT3 expression upon treatment with JPX-0750 at indicated doses, normalized to Actin as loading control. **C**, Western blot analysis for pY-STAT5, t-STAT5, PIM1 and BCL-2 in KOPT-K1 T-ALL cells after treatment with STAT5 degrader JPX-0750 at indicated concentrations or vehicle (Veh.) for 24 h. Actin was used as loading control. **D**, Representative dose-response curve for T-ALL cell lines or controls treated with fostamatinib or gusacitinib at indicated concentrations. Three independent experiments in technical triplicates were performed. Error bars show mean +/-SEM. **E**, Western blot analysis of KOPT-K1 and DND-41 cells, treated with DMSO or 0.5, 2 or 5 µM fostamatinib or gusacitinib for 24 h, showing expression of pY-ZAP70, t-ZAP70, pY-STAT5 and STAT5, α-Tubulin served as loading control. One representative out of two independent experiments is shown. **F**, Dose-response curve and IC_{50} values of *ZAP70* knock-out KOPT-K1 cells vs. controls, in technical triplicates and using two guide RNAs targeting *ZAP70*. **G**, Percent survival of non-leukemic control and T-ALL cells of primary patient samples treated 24 h with fostamatinib at 100, 1000 or 10,000 nM. Mean values of respective concentration vs. DMSO control in 2 technical replicates are shown. Relapsed patients are marked in red. In **D** and **G**, error bars show mean +/-SEM.

Suppl. Fig. 11

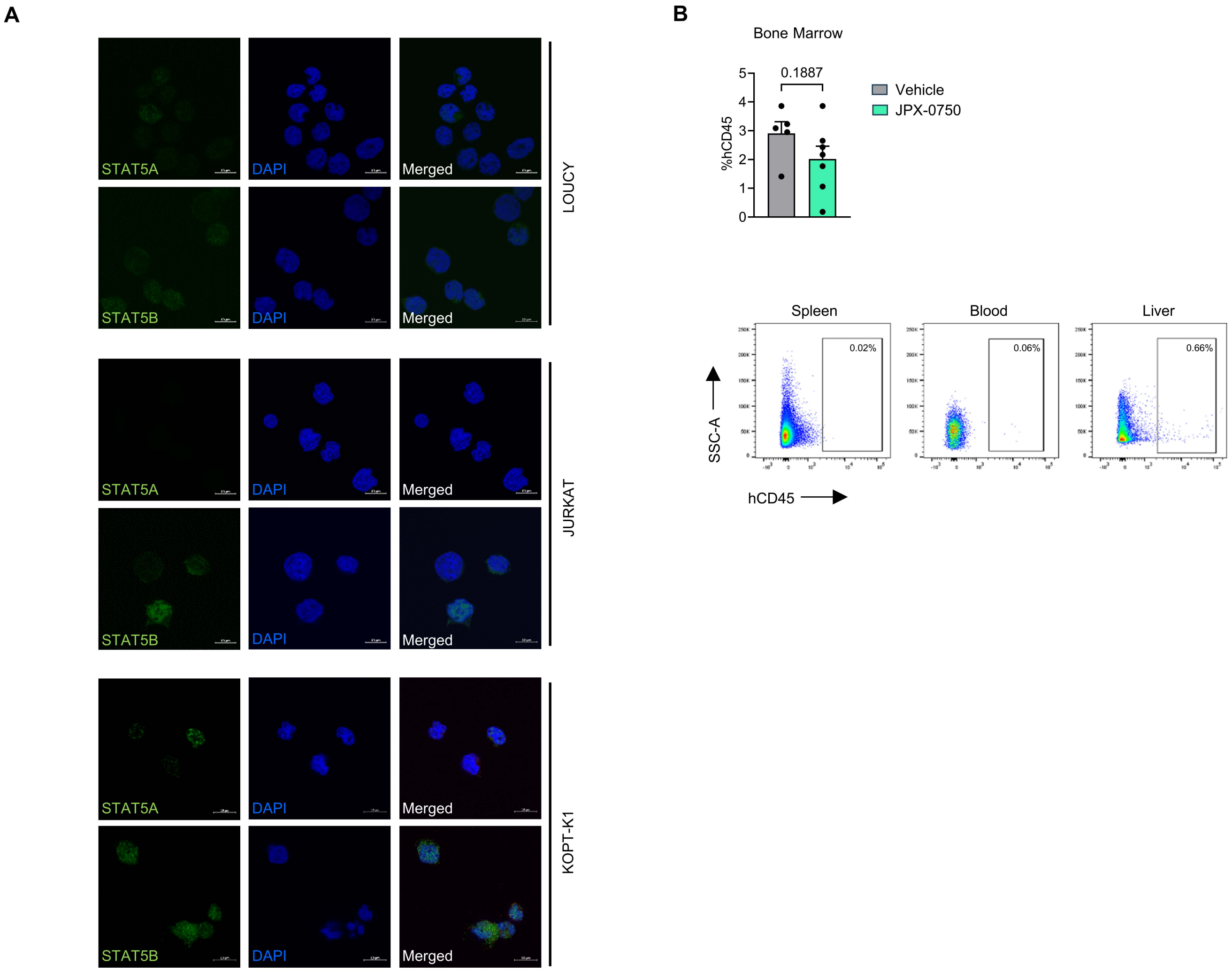

Supplemental Figure S12. STAT5A and STAT5B are present in the nuclei of T-ALL cells. A, Confocal microscopy of LOUCY, JURKAT and KOPT-K1 T-ALL cells stained with antibodies against STAT5A or STAT5B (green) and DAPI (blue). **B**, hCD45⁺ fractions of bone marrow from mice injected with DND-41 cells and treated with either JPX-0750 (n = 7) or vehicle (n = 5), and representative flow cytometry plots of hCD45⁺ fractions in the spleen, blood and liver. In **B**, comparison was done by unpaired two-tailed Student's t-test. Error bars show mean +/-SEM.

Supplemental Tables

GS DETAILS	SIZE	ES	NES	NOM p-val	FDR q-val	FWER p-val	
IFN RESPONSE	50	0.74	2.69	0	0	0	DN
CD8 CYTOTOXIC	36	0.71	2.4	0	0	0	
CD8 CYTOKINE	49	0.61	2.2	0	0	0	
CD4 NV/CM REST	40	0.54	1.88	0	0.001	0.002	
CD4/CD8	46	0.5	1.81	0.001	0.001	0.004	
TREG	48	0.48	1.74	0.004	0.003	0.015	
IFN RESPONSE	50	0.85	3.08	0	0	0	DP
CD8 CYTOKINE	42	0.81	2.85	0	0	0	
CD8 CYTOTOXIC	28	0.75	2.42	0	0	0	
TREG	48	0.55	2.01	0	0.001	0.002	
CD4/CD8	42	0.55	1.9	0	0.001	0.004	
CD4 NV/CM	45	0.4	1.42	0.047	0.067	0.246	
PROLIFERATION	45	0.31	1.09	0.345	0.339	0.849	
+							
CD8 CYTOTOXIC	32	0.78	2.62	0	0	0	SP8
CD8 CYTOKINE	44	0.71	2.58	0	0	0	
IFN RESPONSE	49	0.64	2.36	0	0	0	
CD4/CD8	45	0.49	1.79	0.002	0.003	0.009	
TREG	48	0.38	1.37	0.066	0.1	0.304	
CD4 NV/CM	41	0.3	1.08	0.343	0.341	0.827	

Supplemental Table 1. Correlation analysis by GSEA between RNA-seq of DN, DP and SP8 cells of $STAT5B^{N642H}$ vs. WT and characteristic signatures of T-cell subsets, further described in reference 7 (**Supplemental Methods**). Number of genes in each gene set (SIZE), enrichment score (ES), normalized enrichment score (NES), nominal *P* value (NOM *P*), false discovery rate, q value (FDR q-val) and family-wise error rate, q value (FWER q-val) are shown.

Patient #	Sex	Age at sampling	Disease stage	Sample type	Markers used in screen	Control population	T-ALL population
1	f	79	primary diagnosis	BM	CD3, CD5, CD7	$CD19^+$	$CD3^+$ $CD5^+$ $CD7^+$
2	f	40	primary diagnosis	BM	CD3, CD5, CD7	$CD19^+$	$CD3^+$ $CD7^+$
3	f	21	primary diagnosis	PB	CD3, CD5, CD7	$CD14^+$	$CD5^+$
4	m	40	primary diagnosis	PB	CD3, CD5, CD7	$CD14^+$	$CD3^+$ $CD5^+$ $CD7^+$
5	f	31	primary diagnosis	BM	CD3, CD5, CD7	$CD19^+$	$CD3^+$ $CD5^+$ $CD7^+$
6	m	20	primary diagnosis	BM	CD3, CD5, CD7	$CD19^+$	$CD3^+$
7	m	60	primary diagnosis	BM	CD3, CD5, CD7	$CD19^+$	$CD3^+$ $CD7^+$
8	m	39	primary diagnosis	BM	CD3, CD5, CD7	$CD19^+$	$CD7^+$
9	m	57	primary diagnosis	BM	CD34, CD7	$CD19^+$	$CD34^+$ $CD7^+$
10	m	75	primary diagnosis	BM	CD3, CD5, CD7	$CD19^+$	$CD3^+$ $CD5^+$ $CD7^+$
11	m	62	primary diagnosis	BM	CD1A, CD5, CD7	$CD19^+$	$CD1A^+$ $CD5^+$ $CD7^+$
12	m	41	1st relapse	BM	HLA-DR, CD5, CD3	$CD19^+$	$HLA\text{-}DR^+$ $CD5^+$ $CD3^+$
13	m	31	relapse	PB	CD1A, CD8A, CD3	$CD19^+$	$CD1A^+$ $CD8A^+$
14	m	28	1st relapse	BM	CD3, CD5, CD7	$CD19^+$	$CD3^-$ $CD5^-$ $CD7^-$
15	m	37	1st relapse post HSCT	PB	CD3, CD5, CD7	$CD19^+$	$CD5^+$ $CD7^+$

Supplemental Table 2. Additional clinical information on T-ALL patients, indicating sex, age and disease stage (HSCT: hematopoietic stem cell transplantation) of the patients, sample origin (BM: bone marrow, PB: peripheral blood), surface markers that were used for the screen and populations defining healthy control cells and T-ALL cells.

Supplemental Methods

RNA-seq data processing and analysis

Library preparation and sequencing were performed at the Center for Molecular Medicine (CeMM), Vienna, Austria. Libraries were prepared using the NEBNext ®Ultra^{TM}RNA Library Prep Kit. Next, the samples were multiplexed and sequenced on an Ilumina Hisat2000 sequencer. Quality control was performed using FastQC (version 0.11.8). Removal of low-quality bases and adapter trimming was achieved with trimmomatic, version 0.35 [1], followed by mapping using STAR aligner, version 2.7 [2], with the GRCm38 genome (ftp.ebi.ac.uk/pub/databases/gencode/Gencode_mouse/release_M25/GRCm38.primary_assembly.genome.fa.gz) and ensembl gene annotation release 93 (ftp.ensembl.org/pub/release-93/gtf/mus_musculus/Mus_musculus.GRCm38.93.gtf.gz). The genome for mapping with STAR was generated with the command:

STAR --runMode genomeGenerate --genomeDir STAR93 –genomeFastaFiles GRCm38.primary_assembly.genome.fa –sjdbGTFfile Mus_musculus.GRCm38.93.gtf.

For further analysis, only uniquely mapped reads were considered by using bamtools [3] with the command:

bamtools filter -tag NH:1 <mapped.bam> <filtered.bam>.

For RPKM calculations, the length of a gene was determined by summing up the width of the exons of a gene using the methods sum() and width(). Differential expression analysis contrasting each pair of conditions was performed using DESeq2 [4]. Gene ontology (GO) term enrichment was assessed for "biological process" using the GO Enrichment Analysis Tool at http://geneontology.org [5,6]. For GSEA gene lists obtained from DESeq2 analysis were ranked by fold change and subjected to the GSEAPreranked tool provided by the Broad Institute, version GSEA_Linux_4.0.2. The gene sets from single-cell RNA-seq data of human

T-cells were taken from Szabo et al., 2019 [7]. The ETP-ALL gene sets were obtained as follows: microarray data (Table_S24_12SJETP_40SJnonETP_limma.xlsx) from Zhang et al. 2012 [8] was filtered for probes with refseq ID. The top upregulated genes between ETP-ALL and non-ETP-ALL were determined by ranking via the T-statistic column. Top ranking microarray probes that target different regions of the same gene were not double counted. Accordingly, the number of top probes was set to yield the desired number of genes. Thus, the top 352 microarray probes cover 250 genes. The TCR signaling gene set was compiled from the MSigDB and literature research.

The Venn Diagram was visualized with nvennR [9]. Differentially expressed genes with $P<0.01$ in STAT5B^{N642H} vs. WT contrasts of DN, DP and SP8 cells were intersected. Up- and downregulated genes common to all contrasts were subjected to GO analysis (see above).

Immunohistochemistry and histologic analysis

Mouse organs were incubated 24 h in 4% phosphate-buffered formaldehyde solution (Roti-Histofix; Carl Roth) on a rotator at 4°C, dehydrated, embedded, and cut (4-μm-thick sections). For immunohistochemical staining, heat-mediated antigen retrieval was performed in citrate buffer at pH 6.0 (Dako) and stained with antibodies against CD3 (Thermo Fisher Scientific; RM-9107-S0; dilution 1:300), Ki67 (Novocastra, Leica Biosystem; NCL-Ki67p; dilution 1:1000), TdT (eBioscience; 14-9739-82; dilution 1:50) and pY-ZAP70 (Cell Signaling Technology, 2701, 1:400) using standard protocols. Images were taken using an Olympus BX 53 LED light microscope with an Olympus SC50 camera or a Leica DMi8 with a Leica DMC 2900 camera. Images were analyzed using the ImageJ (version 1.53a) software.

ChIP-seq data processing and analysis

Preprocessing of the raw sequence reads was done with PRINSEQ-lite [10] (version 0.20.4). The remaining high quality reads were aligned with BWA [11] (version 0.7.15-r1140) against the human reference genome (GRCh38) and further processed to bam files with samtools [12] (version 1.4). The deepTools [13] (version 3.5.1) bamCompare function was applied to

create CPM normalized bigWig files. These were then visualized in R [14] (version 4.2.1) with rtracklayer [15] (version 1.56.1). ChIP-seq peaks were called with MACS27 (version 2.1.0) against the respective input controls.

Confocal microscopy

Cultivated cells were washed with PBS and, after settling, fixed with 4% PFA (Roti-Histofix, Carl Roth) on 8 mm coverslips for 10 min. They were permeabilized with 0.5% Triton X-100 (Thermo Scientific) in PBS for 8 min and blocked with 3% BSA (Carl Roth) and 0.1% Triton X-100 in PBS for 1 h at room temperature. The cells were incubated with primary antibodies (**Supplemental Table 7**) for 1 h at room temperature or 4°C overnight. After that, the cells were incubated with secondary antibodies (AlexaFluor^{TM} 488 donkey anti-mouse IgG, Invitrogen, A21202, 1:500) for 1 h at room temperature and stained with DAPI (10 µL/mL) for 10 min. The coverslips were inverted onto a glass slide with a drop of antifade reagent (ProLong^{TM} Gold Antifade Mountant) and imaged on a Zeiss CLSM 880 confocal microscope. Detection channels were adjusted to show no signal in the negative controls.

FACS antibodies (for murine cells)

Target	Fluorochrome	Clone	Company	Cat. no.
CD19	eFluor450	eBio1D3	Invitrogen	48-0193-82
CD25	APC	PC61.5	Invitrogen	17-0251-82
CD3E	FITC	145-2C11	Biolegend	100306
	PerCP-Cyanine5.5	145-2C11	Invitrogen	45-0031-82
CD4	APC-eFluor 780	RM4-5	Invitrogen	47-0042-82
	FITC	GR1.5	Invitrogen	11-0041-82
	PE-Cyanine7	GK1.5	Invitrogen	25-0041-82
CD44	PE	IM7	Invitrogen	12-0441-82
CD5	PE-Cyanine7	53-7.3	Invitrogen	25-0051-81
CD69	FITC	H1.2F3	Invitrogen	11-0691-82
CD8A	PerCP-Cyanine5.5	53-6.7	Invitrogen	45-0081-82
	PE	53-6.7	Invitrogen	12-0081-82
Ly5.1	PE	A20	Invitrogen	12-0453-82
Ly5.2	APC	104	Invitrogen	17-0454-82
Ter119	PE	TER-119	Invitrogen	12-5921-81
Thy1.2	eFluor450	53-2.1	Invitrogen	48-0902-82
	APC	53-2.1	Invitrogen	17-0902-81
Viability dye	APC-eFluor780		Invitrogen	65-0865-14

FACS antibodies (for human cells)

Target	Fluorochrome	Clone	Company	Cat. no.
CD1a	PE-Cy7	HI149	Biolegend	300122
	APC	HI149	BD Biosciences	559775
CD3	PE	HIT3a	Biolegend	300308
CD5	APC-Cyanine7	L17F12	Biolegend	364010
CD7	FITC	6B7	Biolegend	343104
	PE-Cyanine7	6B7	Biolegend	343114
CD8A	APC	SK1	Biolegend	344722
CD14	APC	PC61.5	Biolegend	301808
CD19	APC	HIB19	Biolegend	302211
	FITC	SJ25C1	Biolegend	363008
CD34	APC	8G12	BD Biosciences	1196918
Viability dye	DAPI		Biolegend	422801

Supplemental Table 3. Antibodies and dyes used for FACS measurement and sorting, Cat. no.: catalogue number

Western blot antibodies

Target	Dilution	Clone	Company	Cat. no.
t-STAT5	1:1000	89/Stat5	BD Biosciences	610191
BCL-2	1:1000	124	Cell Signaling Technology	15071S
PIM1	1:1000	D8D7Y	Cell Signaling Technology	54523S
CD3zeta	1:500	6B10.2	Santa Cruz Biotechnology	sc-1239
t-ZAP70	1:1000	521626	R&D Systems	MAB3709
t-FYN	1:750	361228	R&D Systems	MAB3574
t-GRAP2	1:1000	Polyclonal	R&D Systems	AF4640

t-STAT3	1:1000	84/Stat3	BD Biosciences	610189
pY-STAT5 (Tyr694/699)	1:1000	Polyclonal	Invitrogen	71-6900
pY-ZAP70 (Tyr319)	1:1000	65E4	Cell Signaling Technology	2717
pY-SRC (Tyr416)	1:1000	D49G4	Cell Signaling Technology	6943
pY-PLCγ1 (Tyr783)	1:1000	D6M9S	Cell Signaling Technology	14008
pY-STAT3 (Tyr705)	1:1000	Polyclonal	Cell Signaling Technology	9131
α-Tubulin	1:5000	DM1A	Santa Cruz Biotechnology	sc-32293
Actin	1:5000	Polyclonal	Santa Cruz Biotechnology	sc-1615

Supplemental Table 4. Antibodies used for Western blot analysis, Cat. no.: catalogue number

ChIP antibodies

**Target**	**Company**	**Cat. no.**
STAT5B	R&D Systems	AF1584
STAT5B	Invitrogen	13-5300

Supplemental Table 5. Antibodies used for ChIP-seq analysis, Cat. no.: catalogue number

**Target_#**	Forward sequence	Reverse sequence
STAT5B_1	caccgGTTCATTGTACAATATATGG	aaacCCATATATTGTACAATGAACc
STAT5B_2	caccgAGCATTGTCCCAGAGAACAG	aaacCTGTTCTCTGGGACAATGCTc
ZAP70_1	caccgGCAGAAGATGAAAGGGCCGG	aaacCCGGCCCTTTCATCTTCTGCc
ZAP70_2	caccgGTTAACCAGCAGGACGTTGC	aaacGCAACGTCCTGCTGGTTAACc
AAVS1	caccGCTGTGCCCCGATGCACAC	aaacGTGTGCATCGGGGCACAGC
RPL17	caccGACATCTTTCAGATACTTCG	aaacCGAAGTATCTGAAAGATGTC

Supplemental Table 6. Forward and reverse sequences of guide RNAs used for CRISPR/*Cas9*-mediated knock-out experiments

**Target**	**Dilution**	**Clone**	**Company**	**Cat. no.**
STAT5A	1:500	C-6X	Santa Cruz Biotechnology	sc-271542
STAT5B	1:500	G-2X	Santa Cruz Biotechnology	sc-1656

Supplemental Table 7. Antibodies used for confocal microscopy, Cat. no.: catalogue number

Additional references

1. Bolger AM, Lohse M, Usadel B. Trimmomatic: a flexible trimmer for Illumina sequence data. Bioinformatics. 2014;30:2114–20.

2. Dobin A, Davis CA, Schlesinger F, Drenkow J, Zaleski C, Jha S, et al. STAR: ultrafast universal RNA-seq aligner. Bioinformatics. 2013;29:15–21.

3. Barnett DW, Garrison EK, Quinlan AR, Strömberg MP, Marth GT. BamTools: a C++ API and toolkit for analyzing and managing BAM files. Bioinformatics [Internet]. 2011;27:1691–2. Available from: https://doi.org/10.1093/bioinformatics/btr174

4. Love MI, Huber W, Anders S. Moderated estimation of fold change and dispersion for RNA-seq data with DESeq2. Genome Biol. 2014;15:550.

5. Ashburner M, Ball CA, Blake JA, Botstein D, Butler H, Cherry JM, et al. Gene ontology: tool for the unification of biology. The Gene Ontology Consortium. Nat Genet. 2000;25:25–9.

6. Mi H, Muruganujan A, Ebert D, Huang X, Thomas PD. PANTHER version 14: more genomes, a new PANTHER GO-slim and improvements in enrichment analysis tools. Nucleic Acids Res. 2019;47:D419–26.

7. Szabo PA, Levitin HM, Miron M, Snyder ME, Senda T, Yuan J, et al. Single-cell transcriptomics of human T cells reveals tissue and activation signatures in health and disease. Nat Commun. 2019;10:4706.

8. Zhang J, Ding L, Holmfeldt L, Wu G, Heatley SL, Payne-Turner D, et al. The genetic basis of early T-cell precursor acute lymphoblastic leukaemia. Nature. 2012;481:157–63.

9. Pérez-Silva JG, Araujo-Voces M, Quesada V. nVenn: generalized, quasi-proportional Venn and Euler diagrams. Bioinformatics. England; 2018;34:2322–4.

10. Schmieder R, Edwards R. Quality control and preprocessing of metagenomic datasets. Bioinformatics. 2011;27:863–4.

11. Li H, Durbin R. Fast and accurate short read alignment with Burrows-Wheeler transform. Bioinformatics. 2009;25:1754–60.

12. Li H, Handsaker B, Wysoker A, Fennell T, Ruan J, Homer N, et al. The Sequence Alignment/Map format and SAMtools. Bioinformatics. 2009;25:2078–9.

13. Ramírez F, Dündar F, Diehl S, Grüning BA, Manke T. deepTools: a flexible platform for exploring deep-sequencing data. Nucleic Acids Res. 2014;42:W187-91.

14. Team RC. A Language and Environment for Statistical Computing. R Found Stat Comput. 2015;

15. Lawrence M, Gentleman R, Carey V. rtracklayer: an R package for interfacing with genome browsers. Bioinformatics. 2009;25:1841–2.
