# Supplementary material for: Hyperactive STAT5 hijacks T cell receptor signaling and drives immature T cell acute lymphoblastic leukemia
Source: J Clin Invest. 2024 Apr 15;134(8):e168536. doi: 10.1172/JCI168536 (PMC11014662; doi:10.1172/JCI168536)

Uncropped Western blot membranes

all further informations (clone, dilution, catalogue number, company can be found in the Supplemental methods

Figure 3

B

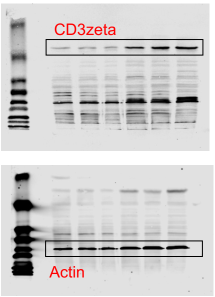

D

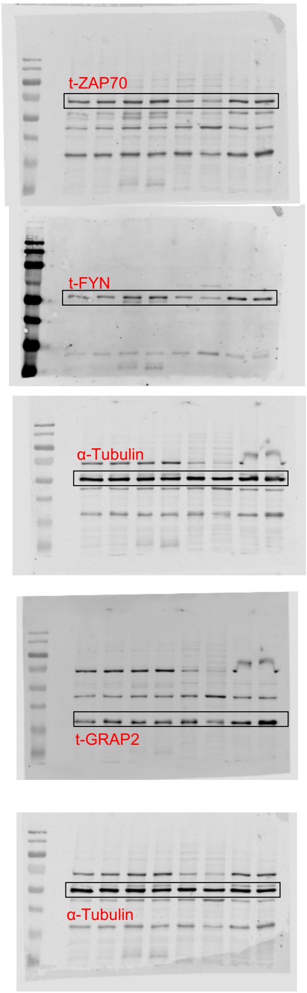

Figure 5A

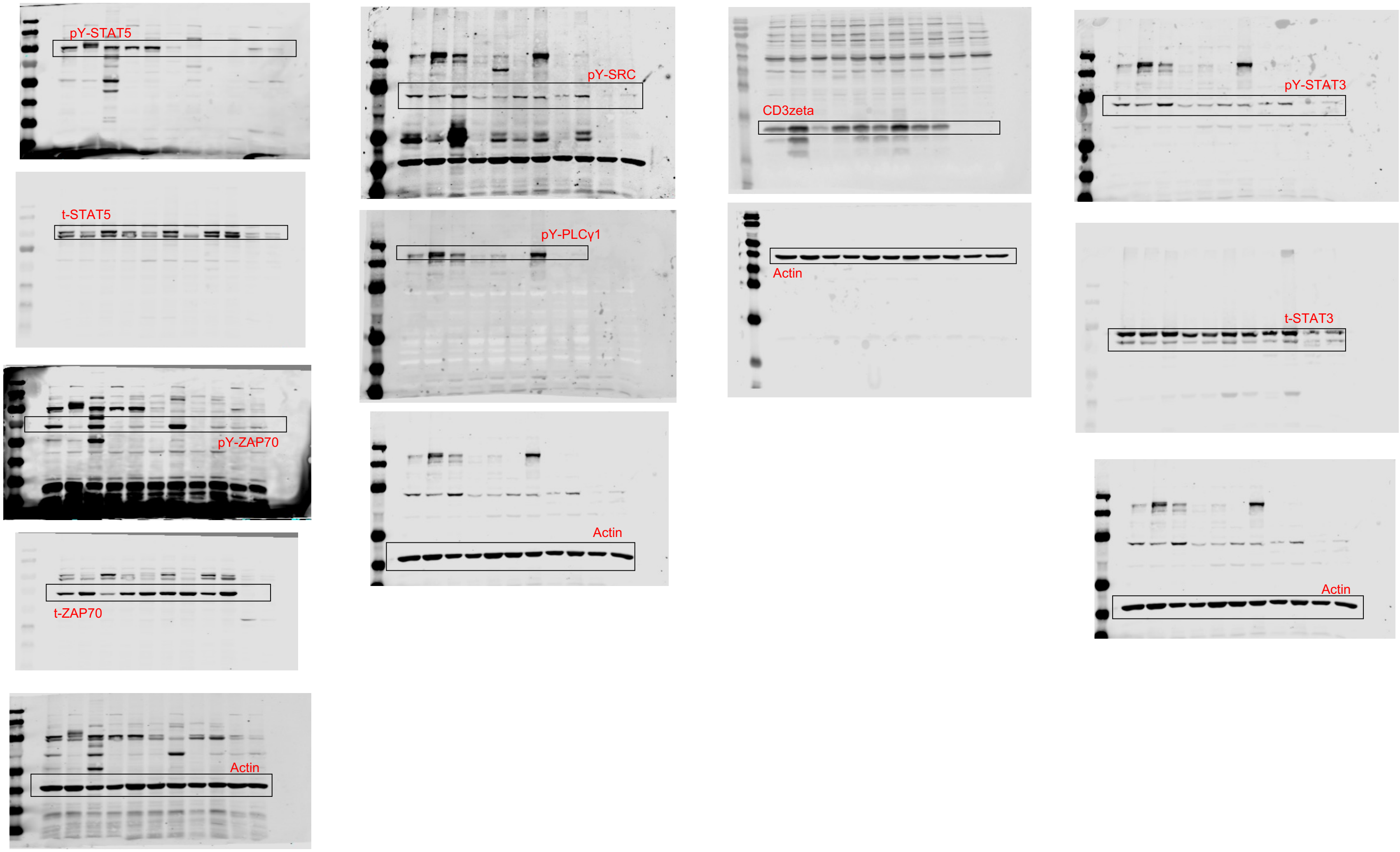

pY-STAT5
t-STAT5
pY-ZAP70
t-ZAP70
Actin
pY-SRC
pY-PLCγ1
Actin
CD3zeta
Actin
pY-STAT3
t-STAT3
Actin

Figure 6B

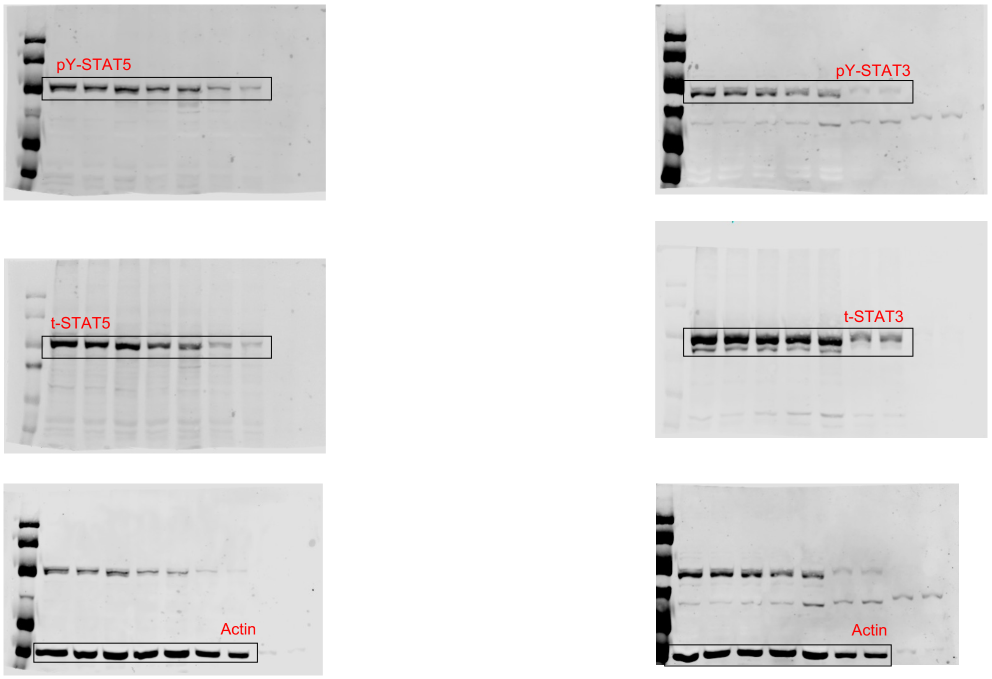

Suppl. Figure 1B

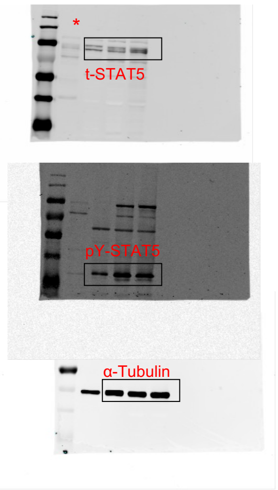

* First lane excluded due to bad sample quality

Suppl. Figure 9B

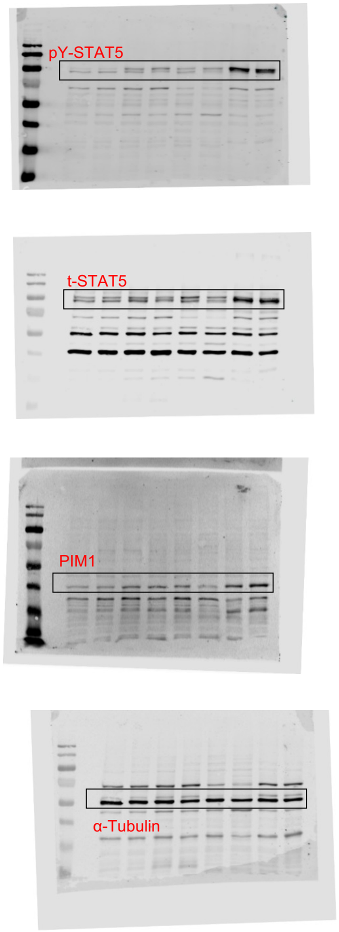

Suppl. Figure 11C

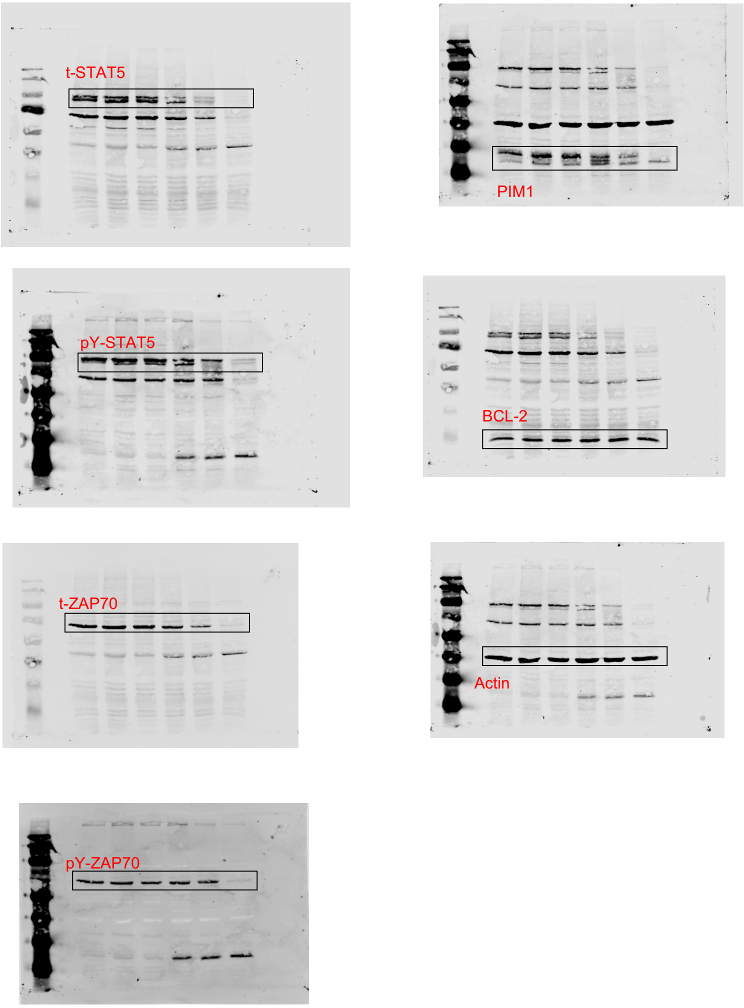

Suppl. Figure 11E

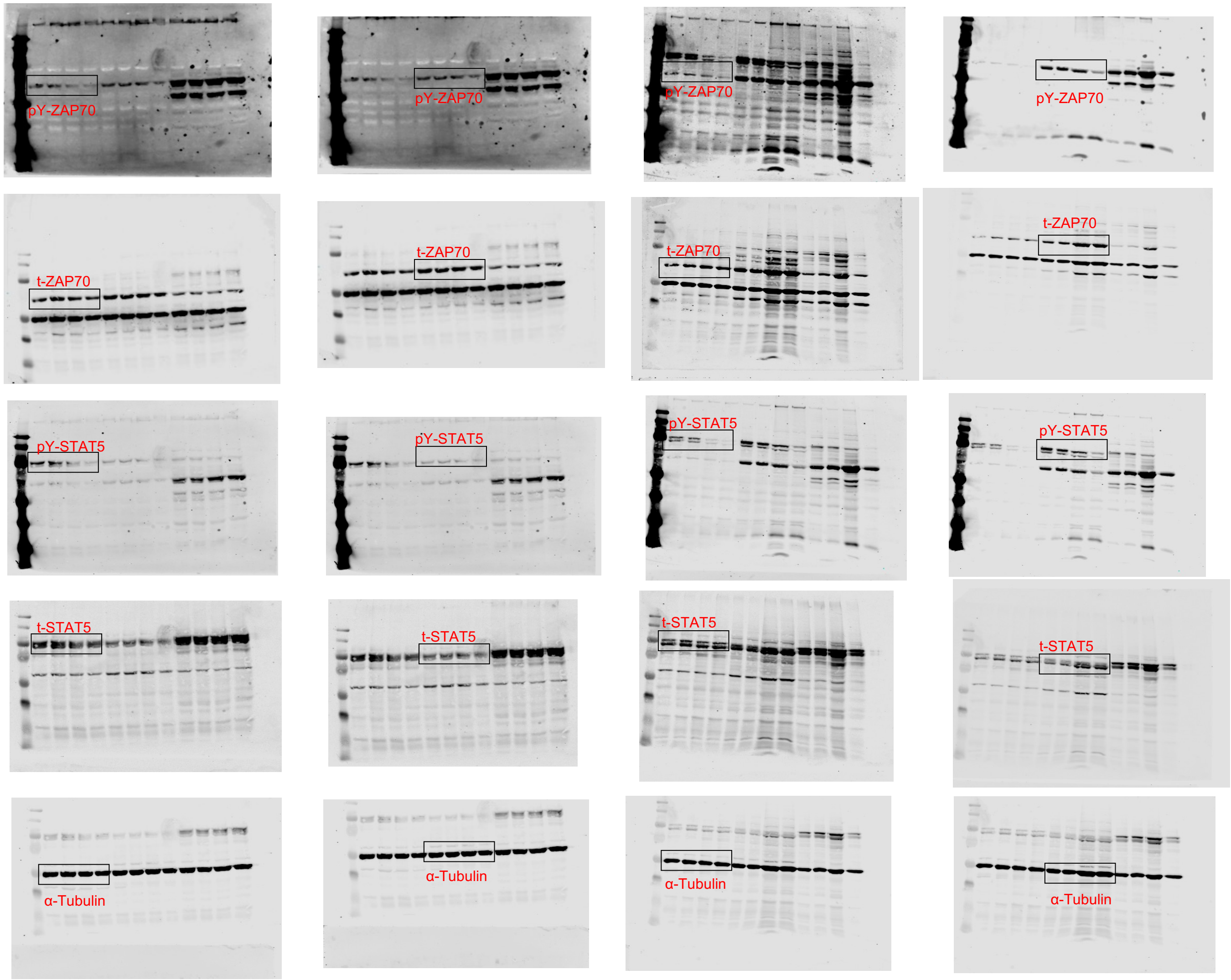

Supplement: Unedited blot and gel images [file jci-134-168536-s149.pdf]
